# Targeting piRNA‐137463 Inhibits Tumor Progression and Boosts Sensitivity to Immune Checkpoint Blockade via *De Novo* Cholesterol Biosynthesis in Lung Adenocarcinoma

**DOI:** 10.1002/advs.202414100

**Published:** 2024-12-18

**Authors:** Yuning Zhan, Fanglin Tian, Weina Fan, Xin Li, Xiangyu Wang, Hongxia Zhang, Xin Hong, Xin Wang, Li Cai, Yang Song, Ying Xing

**Affiliations:** ^1^ The Fourth Department of Medical Oncology Harbin Medical University Cancer Hospital 150 Haping Road Harbin 150081 China; ^2^ NHC and CAMS Key Laboratory of Molecular Probe and Targeted Theranostics Harbin Medical University Harbin 150001 China; ^3^ Imaging Center Harbin Medical University Cancer Hospital Harbin 150081 China; ^4^ The Department of Orthopedics The Second Affiliated Hospital of Harbin Medical University Harbin 150001 China

**Keywords:** cholesterol metabolism, immune escape, lipid raft, lung adenocarcinoma, piRNA‐137463

## Abstract

The important role of PIWI‐interacting RNAs (piRNAs) in tumors has garnered increasing attention. However, research on their role in lung adenocarcinoma (LUAD) remains limited. Elevated levels of piRNA‐137463 have been linked to poor prognosis in LUAD patients. Inhibition of piRNA‐137463 curbed the proliferation, migration, and invasion of LUAD cells, enhanced T cell cytotoxicity through increased IFN‐γ secretion, disrupted cholesterol metabolism, and reduced intracellular cholesterol, lipid raft content, and PD‐L1 expression in LUAD cells. Bioinformatic prediction identified a potential interaction between piRNA‐137463 and lncRNA LOC100128494. Inhibiting piRNA‐137463 increased the stability and expression of LOC100128494, which further modulated insulin‐induced gene 1 protein (INSIG1) levels via a competitive endogenous RNA network involving LOC100128494 and miR‐24‐3p. Notably, the effect of piRNA‐137463 in LUAD cells is dependent on the expression of LOC100128494 and INSIG1. Inhibiting the expression of piRNA‐137463 with AntagopiRNA‐137463 suppressed tumor growth and metastasis via LOC100128494 in nude mice and enhanced the response of LUAD to anti‐PD‐1 therapy in immune‐competent mice. In summary, this study elucidates the role of piRNA‐137463 in the reprogramming of cholesterol metabolism, which drives the progression of LUAD, thereby identifying a new target for the comprehensive clinical management of LUAD.

## Graphical abstract

In lung adenocarcinoma (LUAD), piRNA‐137463 silences the expression of the cholesterol synthesis inhibitor INSIG1 through the LOC100128494/miR‐24‐3p/INSIG1 axis, thereby enhancing cholesterol biosynthesis. The elevated cholesterol not only increases the content of lipid rafts, which promotes proliferation and metastasis; it also stabilizes PD‐L1 to facilitate immune evasion of the cancer cells, ultimately leading to the progression of LUAD.

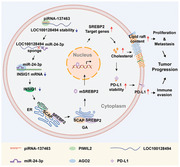

## 1 Introduction

Despite significant advancements in diagnostic and therapeutic techniques, lung cancer continues to be the predominant cause of tumor‐related deaths worldwide according to Global Cancer Statistics 2024.^[^ ^1^ ^]^ Small‐cell lung carcinoma and non‐small‐cell lung carcinoma (NSCLC) represent the two principal subtypes of lung cancer, comprising 15% and 85% of total lung cancer cases, respectively.^[^ ^2^ ^]^ NSCLC is further classified into three main types: lung adenocarcinoma (LUAD), squamous cell carcinoma, and large cell carcinoma.^[^ ^3^ ^]^ LUAD comprises over 40% of all lung cancer cases, with most diagnoses occurring at advanced stages, limiting therapeutic options and often leading to poor outcomes.^[^ ^4^ ^]^ Therefore, a comprehensive understanding of the mechanisms driving LUAD pathogenesis is crucial for developing effective therapeutic strategies.

PIWI‐interacting RNAs (piRNAs) constitute a class of small non‐coding RNAs, typically ranging from 24 to 32 nucleotides in length, that bind to PIWI subfamily proteins.^[^ ^5^ ^]^ Compared to microRNAs (miRNAs), piRNAs are slightly longer and have a characteristic 2′‐O‐methyl modification at their 3′‐end.^[^ ^6^ ^]^ Mechanistically, the piRNA/PIWI complex binds to RNA sequences complementary to the piRNA bases, facilitating the cleavage of these target RNAs through the endonuclease activity of PIWI proteins, thereby promoting the degradation of target RNAs.^[^ ^7^ ^]^ Additionally, piRNAs can suppress the transcription of target genes within the nucleus by recruiting epigenetic and chromatin‐modifying factors, such as DNA methyltransferases.^[^ ^8^ ^]^ piRNAs can also interact with proteins, such as YTH domain family protein 2, to regulate target mRNA expression.^[^ ^9^ ^]^ The PIWI‐piRNA complex is well‐known for its crucial regulatory role in reproductive system development and is typically expressed exclusively in germ cells, with little to no expression in normal somatic tissues.^[^ ^10^ ^]^ However, recent studies have revealed aberrant piRNA expression in various cancers, where they contribute to tumor progression, gaining significant interest as potential targets for precision tumor therapy.^[^ ^7^, ^8^, ^11^ ^]^ For instance, piR‐DQ593109 targets and suppresses LncRNA MEG3, regulating the permeability of the blood‐tumor barrier through the MEG3/miR‐330‐5p/RUNX3 competitive endogenous RNA (ceRNA) mechanism, thereby limiting the effective delivery of chemotherapeutic agents to gliomas.^[^ ^7c^ ^]^ piRNA‐651 downregulates phosphatase and tensin homolog deleted on chromosome ten expression by recruiting DNA methyltransferase 1 to its promoter region and facilitating methylation, thereby promoting the proliferation and migration of breast cancer cells.^[^ ^8a^ ^]^

Previous studies have demonstrated that piRNAs hold significant promise as potential prognostic biomarkers and therapeutic targets for LUAD as well as NSCLC.^[^ ^11^, ^12^ ^]^ piR‐651 is elevated in lung cancer tissues, promoting metastasis and viability in LUAD cells while reducing G0/G1 phase arrest.^[^ ^13^ ^]^ The RASSF1C‐PIWIL1‐piRNA signaling axis, which includes piR‐52200, piR‐35127, piR‐34871, and piR‐46545, plays a crucial role in modulating lung cancer cell proliferation, invasion, migration, and apoptosis.^[^ ^14^ ^]^ In contrast, some piRNAs function as tumor suppressors. piR‐55490 promotes the degradation of mammalian targets of rapamycin via direct interaction, thereby diminishing tumor cell proliferation.^[^ ^15^ ^]^ Another tumor suppressor, piR‐211106, reverses cisplatin resistance in LUAD cells via interaction with pyruvate carboxylase.^[^ ^16^ ^]^ Notably, piRNAs are more stable due to their protective 2′‐O‐methylation modification at the 3′ ends, which shield them from nucleolytic degradation.^[^ ^17^ ^]^ A diagnostic panel consisting of two piRNAs, piR‐hsa‐26925 and piR‐hsa‐5444, was found to be elevated in LUAD patients compared to healthy controls. This panel exhibited excellent diagnostic performance for LUAD, highlighting its potential utility in clinical settings.^[^ ^18^ ^]^

Metabolic reprogramming is a hallmark of cancer, enabling cancer cells to meet their growing needs by altering metabolic patterns and creating favorable conditions for malignant behaviors.^[^ ^19^ ^]^ Among various metabolic pathways, dysregulated lipid metabolism plays a pivotal role in maintaining cellular homeostasis and signaling.^[^ ^20^ ^]^ In particular, disruptions in cholesterol metabolism have been linked to the progression of several tumors, including LUAD.^[^ ^21^ ^]^ Cancer cells often sustain their proliferation, migration, invasion, and immune evasion by increasing cholesterol uptake and synthesis.^[^ ^22^ ^]^ The mature sterol regulatory element‐binding protein 2 (mSREBP2) is an essential transcriptional regulator of genes related to *de novo* cholesterol biosynthesis and uptake.^[^ ^23^ ^]^ The activation of full‐length SREBP2 (flSREBP2) and the formation of mSREBP2 are controlled by insulin‐induced gene 1 protein (INSIG1).^[^ ^24^ ^]^ INSIG1 modulates the post‐translational cleavage of SREBP2 by forming an INSIG‐SCAP‐SREBP2 complex with SREBP cleavage‐activating protein (SCAP), which retains flSREBP2 in the endoplasmic reticulum (ER).^[^ ^24b^ ^]^ Upon a reduction in cholesterol levels or decreased INSIG expression, INSIG dissociates from SCAP, enabling the transport of flSREBP2 to the Golgi apparatus, where proteolytic cleavage generates mSREBP2.^[^ ^23^, ^25^ ^]^

This study aims to identify aberrantly expressed piRNAs that can predict poor survival outcomes for LUAD patients and to elucidate their roles and mechanisms in the development of LUAD. We identified piRNA‐137463 as a key driver of proliferation, invasion, migration, and immune evasion in LUAD. Notably, targeting piRNA‐137463 expression demonstrated a synergistic anti‐cancer effect with anti‐Programmed Death 1 (PD‐1) drugs in LUAD animal models. Mechanistically, we found that inhibiting piRNA‐137463 suppresses cholesterol biosynthesis and inhibits malignant phenotype transformation by regulating the LOC100128494/miR‐24‐3p/INSIG1 axis. Our findings reveal a novel mechanistic link between piRNAs, cholesterol metabolism, tumor progression, and antitumor immunity. These results suggest that targeting piRNA‐137463 or the cholesterol biosynthetic pathway may serve as a promising therapeutic strategy for LUAD.

## 2 Results

### 2.1 piRNA‐137463 is Elevated in LUAD and is a Negative Prognostic Factor for LUAD Patients

**Figure 1:**
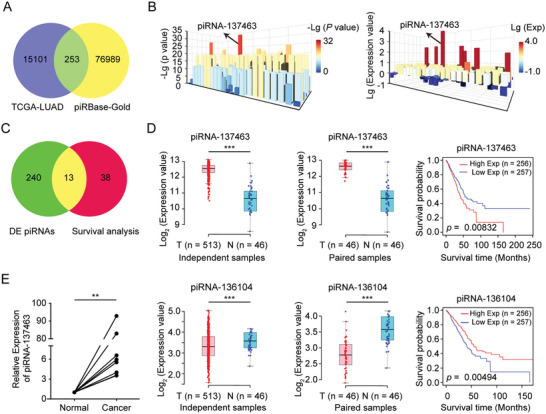
Identification of survival‐associated gold standard piRNAs in LUAD. A) A Venn diagram showing that 253 piRNAs were identified as gold standard, LUAD‐specific, and differentially expressed in paired LUAD tissues compared to their matched normal tissues, based on the TCGA and piRBase databases. B) *P*‐values and relative expression levels of the 253 differentially expressed piRNAs in LUAD tissues relative to matched normal tissues. C) A Venn diagram showing the differentially expressed and prognosis‐related piRNAs in LUAD from the TCGA database. D) Differential expression of piRNA‐137463 and piRNA‐136104 in LUAD and normal tissues (left one‐third panel) and in LUAD‐matched normal tissues (left two‐thirds panel). Survival curves for piRNA‐137463 and piRNA‐136104 in LUAD patients (right one‐third panel). E) Detection of piRNA‐137463 expression in 8 pairs of LUAD tissues and their paired normal tissues by qPCR. Statistics analyses were performed using paired t‐tests (E) unpaired t‐tests and log‐rank tests (D). ^**^ *P* < 0.01, ^***^ *P* < 0.001.

To identify potential oncogenic or tumor‐suppressive piRNAs in LUAD, we examined the expression of 77569 human‐specific, gold‐standard piRNAs from piRBase in the TCGA‐LUAD dataset. Gold‐standard piRNAs are recognized for their high functionality and reliability in critical biological processes, exhibiting 2′‐O‐methylation modification at the 3′ end and capable of binding to PIWI proteins.^[^ ^26^ ^]^ In the TCGA‐LUAD dataset, 15354 piRNAs were specifically and differentially expressed in paired LUAD tissues compared to normal tissues, including 253 piRBase gold standard piRNAs (**Figure** **1**A). Among them, piRNA‐137463 had the lowest *P*‐value and the highest expression level in LUAD tissues (Figure 1B). Survival analysis indicated that 51 piRNAs were associated with patient survival in LUAD, 13 of which were differentially expressed in LUAD (Figure 1C). Consistency analysis between prognosis and differential expression, where high expression in tumors predicts poorer survival and low expression predicts better survival, identified piRNA‐137463 and piRNA‐136104 as candidate genes (Figure 1D). Due to its higher gene expression value, piRNA‐137463 was selected as the better candidate between the two genes for follow‐up studies. Quantitative real‐time PCR (qPCR) analysis of eight matched pairs of LUAD and normal tissues demonstrated that piRNA‐137463 was significantly overexpressed in LUAD samples compared to the normal tissues (Figure 1E). Therefore, we postulated that piRNA‐137463 may facilitate the progression of LUAD, contributing to poorer survival outcomes for LUAD patients.

### 2.2 Inhibition of piRNA‐137463 Impedes Proliferation, Metastasis, and Immune Evasion of LUAD Cells

**Figure 2:**
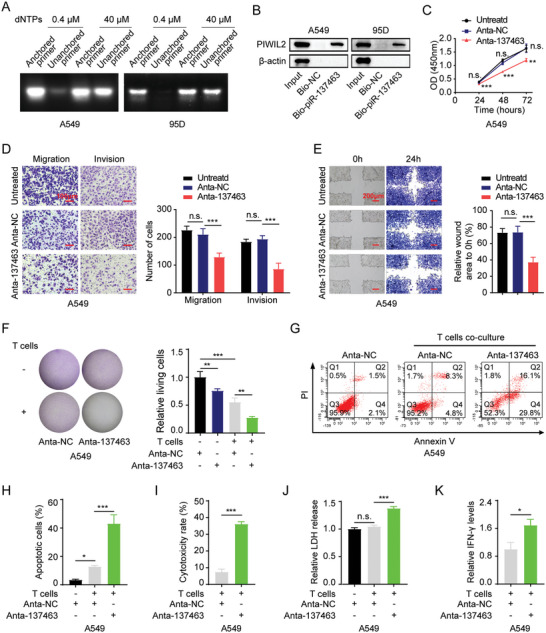
Suppression of piRNA‐137463 reduces proliferation, metastatic potential, and immune escape of LUAD cells. A) The 2′‐O‐methylation at the 3′ end of piRNA‐137463 was verified by RTL‐P assays. B) Biotin‐labeled piRNA pull‐down assays to detect the binding of piRNA‐137463 and PIWIL protein. C) Reduced proliferative capacity of A549 cells following piRNA‐137463 silencing, analyzed by CCK‐8 assays. D, E) The effects of piRNA‐137463 inhibition on the invasive and migratory abilities of A549 cells were analyzed by transwell assays (D) and wound healing assays (E). F) Crystal violet staining images (left) and quantification (right) of A549 cells co‐cultured with activated T cells in Anta‐NC and Anta‐137463. G, H) T cell‐mediated apoptosis of A549 cells detected by flow cytometry in Anta‐NC and Anta‐137463. I) Effect of piRNA‐137463 inhibition on the efficiency of T cell killing of A549 cells. J) Levels of LDH released from A549 cells co‐cultured with T cells in Anta‐NC and Anta‐137463. K) Levels of IFN‐γ secreted by T cells co‐cultured with A549 cells in Anta‐NC and Anta‐137463. Data are presented as mean ± SD. Statistical analyses were performed using t‐test (I and K), two‐way (C), and one‐way ANOVA (D‐F, H, and J). ^*^ *P *< 0.05, ^**^ *P* < 0.01, ^***^ *P* < 0.001, *n.s*., not significant.

We quantified the expression levels of piRNA‐137463 across six LUAD cell lines using qPCR and identified A549 and 95D cells for subsequent experiments based on their elevated piRNA‐137463 expression (Figure S1A, Supporting Information). To confirm the piRNA characteristics of piRNA‐137463, we employed reverse transcription (RT) at low dNTP concentration followed by PCR (RTL‐P) and biotin‐modified small RNA pull‐down. These analyses confirmed that piRNA‐137463 possesses the essential features of piRNAs: 2′‐O methylation of the 3′ end (**Figure** **2**A) and the ability to interact with the chaperone protein PIWI2 (Figure 2B; Figure S1B, Supporting Information). To enable an evaluation of the function of piRNA‐137463 in LUAD cells, we constructed AntagopiR‐137463 (Anta‐137463), an inhibitor designed to decrease piRNA‐137463 expression. Anta‐137463 successfully reduced its expression compared to cells treated with AntagopiR‐negative control (Anta‐NC) in the A549 and 95D cell lines (Figure S1B, Supporting Information), providing a mean to interrogate the function of piRNA‐137463 in LUAD cells.

As pivotal hallmarks of cancer, persistent proliferation, migration, tissue invasion, and escape of immune destruction drive tumor progression.^[^ ^27^ ^]^ Therefore, we investigated whether piRNA‐137463 contributes to these malignant behaviors. Through long‐term colony formation, Cell Counting Kit‐8 (CCK‐8), and 5‐Ethynyl‐2′‐deoxyuridine (EdU) labeling assays, we found that the inhibition of piRNA‐137463 expression using Anta‐137463 attenuated the proliferative capacity of A549 and 95D cells (Figure 2C; Figure S1C–G, Supporting Information). Transwell and scratch assays revealed that reduced expression of piRNA‐137463 substantially diminished the migratory and invasive abilities of A549 and 95D cells (Figure 2D,E; Figure S2A,B, Supporting Information). Inhibiting piRNA‐137463 expression also sensitized LUAD cells to T cell‐mediated killing in T cell‐mediated antitumor killing assays (Figure 2F; Figure S2C, Supporting Information) and elevated the proportion of apoptotic cancer cells following co‐culture with T cells in flow cytometry (Figure 2G,H; Figure S2D,E, Supporting Information). Moreover, lactate dehydrogenase (LDH) release assays further indicated that the inhibition of piRNA‐137463 increased endogenous LDH release by tumor cells (Figure 2I,J; Figure S2F,G, Supporting Information), accompanied by a remarkable increase in the level of IFN‐γ, an indicator of T cell killing efficiency (Figure 2K; Figure S2H, Supporting Information), suggesting an increase in T‐cell mediated cell death. Taken together, our data suggest that piRNA‐137463 is required for the malignant phenotypes of LUAD cells, including proliferation, invasion, migration, and immune escape.

### 2.3 Silencing of piRNA‐137463 Reduces Cholesterol Levels in LUAD Cells

**Figure 3:**
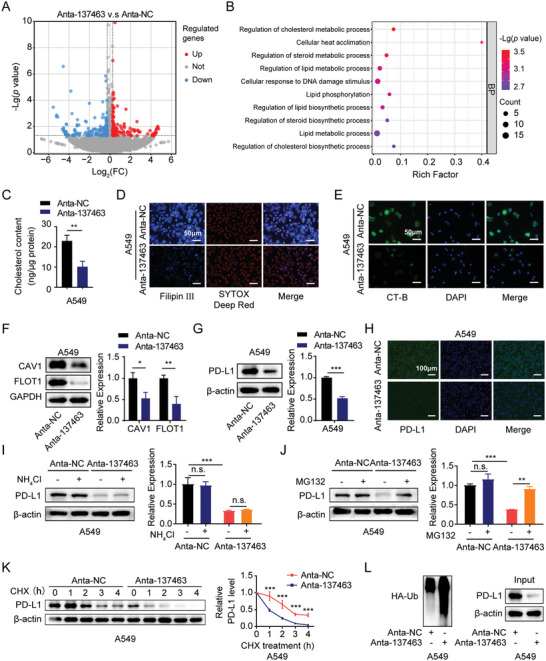
piRNA‐137463 regulates cholesterol metabolism, lipid raft content, and programmed cell death ligand 1 (PD‐L1) expression in LUAD cells. A) Volcano plots of RNA transcriptome sequencing analysis in cells with downregulated piRNA‐137463 versus cells in the control group. B) GO analysis of differentially expressed genes in piRNA‐137463 downregulated cells. C) Effect of piRNA‐137463 downregulation on cholesterol content in A549 cells. D) Filipin III staining of free cholesterol in A549 cells after antagopiR‐137463 treatment. Nuclei were stained with SYTOX Deep Red. E) Effect of antagopiR‐137463 on lipid raft content in A549 cells. Lipid raft content was indicated by fluorescence intensity of cholera toxin subunit B (CT‐B) and nuclei were stained with DAPI. F) Immunoblotting of lipid raft markers in Anta‐NC and Anta‐137463. G,H) The effect of antagopiR‐137463 on PD‐L1 expression was detected by immunoblotting (G) and Immunofluorescence (H). I,J) PD‐L1 expression in Anta‐NC and Anta‐137463 was detected by immunoblotting after treatment with or without 10 mM NH_4_Cl (I) or 10 µm MG132 (J) for 8 h. K) The effect of piRNA‐137463 inhibition on PD‐L1 protein stability was investigated by CHX chase assays. L) AntagopiR‐137463 enhanced the ubiquitin‐mediated degradation of PD‐L1. Cells were transfected with hemagglutinin‐tagged ubiquitin (HA‐Ub) plasmid and treated with MG132 for 8 h. The ubiquitination level of PD‐L1 was detected by immunoprecipitation and immunoblotting. Data are presented as mean ± SD. Statistical analyses were performed using t‐tests (C, F, and G) and two‐way ANOVA (I and J). ^*^ *P* < 0.05, ^**^ *P* < 0.01, ^***^ *P* < 0.001, *n.s*., not significant.

To elucidate the potential mechanisms by which piRNA‐137463 facilitates the malignant behaviors of LUAD cells, we conducted RNA sequencing (RNA‐seq) analysis to examine the gene expression profile of A549 cells treated with either Anta‐137463 or Anta‐NC. As shown in **Figure** **3**A, the DE analysis identified 310 genes differentially expressed following piRNA‐137463 inhibition (|fold change| > 1.2, *P* < 0.05). We then conducted functional enrichment analysis on the differentially expressed genes modulated by piRNA‐137463. Gene ontology (GO) analysis suggested that piRNA‐137463 is involved in cholesterol metabolism, sterol, and lipid biosynthesis (Figure 3B). Kyoto Encyclopedia of Genes and Genomes (KEGG) pathway analysis showed that piRNA‐137463 is associated with fatty acid biosynthesis, miRNAs in cancer, and multiple oncogenic pathways (Figure S3A, Supporting Information). Reactome enrichment analysis implied a role for piRNA‐137463 in cholesterol biosynthesis regulation through SREBP and signaling pathways such as PI3K/AKT (Figure S3B, Supporting Information). Consistent with the results of these functional enrichment analyses, inhibition of piRNA‐137463 significantly reduced cholesterol levels in LUAD cells (Figure 3C; Figure S3C, Supporting Information). Intracellular cholesterol accumulation can be visualized using filipin staining.^[^ ^28^ ^]^ Fluorescence microscopy showed that, compared with control cells, Anta‐137463 treatment resulted in a decrease in cellular filipin III‐bound staining (Figure 3D; Figure S3D, Supporting Information), suggesting a reduced intracellular cholesterol accumulation. These findings suggest that piRNA‐137463 increased cholesterol levels and promoted cholesterol metabolism.

### 2.4 Targeting piRNA‐137463 Decreases Lipid Raft Content and Programmed Cell Death Ligand 1 (PD‐L1) Expression in LUAD Cells

Cholesterol synthesis and metabolism have been recognized as regulators of lipid rafts, which are involved in cancer proliferation, epithelial‐to‐mesenchymal transition (EMT), and metastasis.^[^ ^29^ ^]^ To examine the impact of piRNA‐137463 on lipid raft, we performed lipid raft labeling assays and analyzed the expression of lipid raft markers. Suppression of piRNA‐137463 decreased fluorescently labeled cholera toxin subunit B (CT‐B) staining intensity in A549 and 95D cells (Figure 3E; Figure S3E, Supporting Information), suggesting a reduced lipid raft content. The protein levels of the lipid raft markers caveolin‐1 (CAV1) and flotillin‐1 (FLOT1) were attenuated in the Anta‐137463 group compared to the Anta‐NC group (Figure 3F; Figure S3F, Supporting Information). Our data suggest that piRNA‐137463 plays an important role in the regulation of lipid raft content.

Moreover, cholesterol interacts directly with the transmembrane domain of PD‐L1, facilitating immune evasion by forming a sandwich‐like structure that stabilizes PD‐L1 and protects it from ubiquitination‐mediated proteasomal degradation in cancer cells.^[^ ^30^ ^]^ We hypothesized that silencing piRNA‐137463 might downregulate the expression of PD‐L1 by promoting its proteasomal degradation. Compared with the control treatment, the Anta‐137463 treatment suppressed the expression of PD‐L1 protein (Figure 3G,H; Figure S3G,H, Supporting Information). Proteasome inhibitor MG132, but not lysosomal inhibitor NH_4_Cl, reversed the reduction of PD‐L1 protein levels caused by the inhibition of piRNA‐137463 (Figure 3I,J; Figure S3I,J, Supporting Information), suggesting that the effect of piRNA‐137463 on PD‐L1 expression was dependent on proteasomes. Cycloheximide chase assays revealed that the inhibition of piRNA‐137463 markedly shortened the half‐life of the PD‐L1 protein (Figure 3K; Figure S3K, Supporting Information). Therefore, consistent with our hypothesis, the inhibition of piRNA‐137463 substantially enhanced the ubiquitination of endogenous PD‐L1 (Figure 3L; Figure S3L, Supporting Information), suggesting that piRNA‐137463‐mediated immune escape in LUAD is likely through the regulation of PD‐L1.

### 2.5 piRNA‐137463 Directly Interacts with LOC100128494 and Decreases its Expression

**Figure 4:**
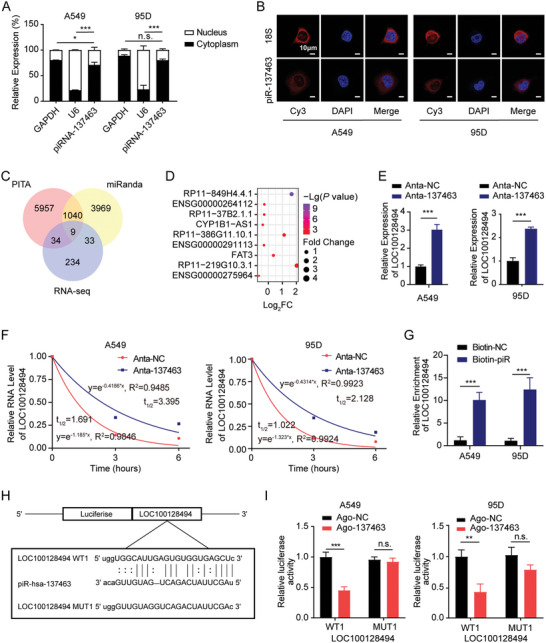
piRNA‐137463 directly interacts with LOC100128494 and reduces its expression in LUAD cells. A) The qPCR assays were used to investigate the subcellular localization of piRNA‐137463 in LUAD cells, with GAPDH and U6 serving as references for cytoplasm and nucleus, respectively. B) FISH assays were conducted to detect the subcellular localization of piRNA‐137463 in A549 and 95D cells. C) Venn diagram illustrating the overlap of PITA miRanda and DE genes from RNA‐seq data for nine RNAs as potential targets of piRNA‐137463. D) Ranking of *P*‐values of potential targets of piRNA‐137463. E) The effect of piRNA‐137463 down‐regulation on LOC100128494 expression in Anta‐NC and Anta‐137463 was detected by qPCR. F) RNA stability of LOC100128494 in Anta‐NC and Anta‐137463. G) Biotin‐labeled piRNA pull‐down assays were used to detect the interaction between piRNA‐137463 and LOC100128494. H) miRanda software predicted the piRNA‐137463 binding site in LOC100128494 (LOC100128494‐WT1 and the designed mutant sequence (LOC100128494‐MUT1) is illustrated. I) The interaction between piRNA‐137463 and LOC100128494 was validated using a dual‐luciferase reporter assay with LOC100128494‐WT1 and LOC100128494‐MUT1 in piRNA‐137463‐overexpressing (Ago‐137463) and control cells (Ago‐NC). Data are presented as mean ± SD. Statistical analyses were performed using one‐way ANOVA (A) and t‐tests (E, G, and I). ^*^ *P* < 0.05, ^**^ *P* < 0.01, ^***^ *P* < 0.001, *n.s*., not significant.

To elucidate the mechanism underlying piRNA‐137463′s actions, we examined its subcellular localization. Nucleocytoplasmic separation experiments and fluorescence in situ hybridization (FISH) suggested that piRNA‐137463 was predominantly expressed in the cytoplasm of LUAD cells (**Figure** **4**A,B). The subcellular localization of piRNA‐137463 implies that it may interact with proteins or RNAs, such as mRNAs and lincRNAs. Given the previously obtained differentially expressed genes from RNA‐seq data following the downregulation of piRNA‐137463, we predicted potential RNA targets of piRNA‐137463 using Probability of Interaction by Target Accessibility (PITA)^[^ ^31^ ^]^ and miRanda^[^ ^11c^ ^]^ (Figure 4C). Among upregulated genes by Anta‐137463 treatment, RP11‐849H4.4.1, also known as LOC100128494, was the most significantly upregulated gene following the silencing of piRNA‐137463, with the smallest *P*‐value (Figure 4D). Meanwhile, qPCR revealed that the LOC100128494 RNA level was increased by the Anta‐137463 treatment compared to the Anta‐NC group (Figure 4E).

piRNAs are recognized for their ability to bind RNAs in a sequence‐specific manner, facilitating RNA degradation through post‐transcriptional gene silencing.^[^ ^32^ ^]^ RNA decay assays revealed that inhibiting piRNA‐137463 enhanced the stability of LOC100128494 in A549 and 95D cells (Figure 4F). Moreover, biotin‐modified small RNA pull‐down assays confirmed the direct interaction between piRNA‐137463 and LOC100128494 (Figure 4G). Subsequently, we employed the miRanda algorithm to predict their interaction sites (Figure 4H). Dual‐luciferase reporter assays showed that agopiR‐137463, an agonist of piRNA‐137463, reduced the luciferase activity of the LOC100128494‐WT1 reporter vector, but not the LOC100128494‐MUT1 reporter vector (Figure 4I). These findings identified LOC100128494 as a target of piRNA‐137463 in LUAD.

### 2.6 LOC100128494 Functions as a ceRNA Regulating the miR‐24‐3p/INSIG1 Axis

**Figure 5:**
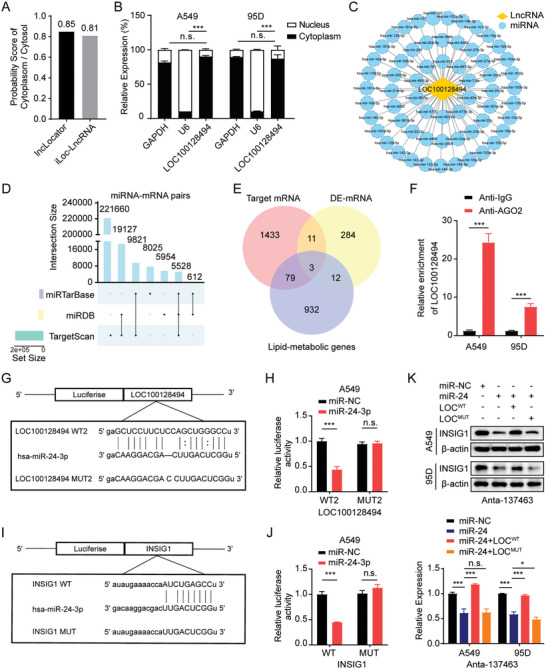
Construction of LOC100128494/miR‐24‐3p/INSIG1 ceRNA network. A) Bar plot illustrating the probability score of subcellular localization of LOC100128494 predicted by lncLocator and iLoc‐LncRNA. B) qPCR analysis investigating the subcellular localization of LOC100128494, with GAPDH and U6 serving as references for the cytoplasm and nucleus, respectively. C) Candidate target miRNAs of LOC100128494 predicted by miRcode. D) Upset plot of miRNA‐mRNA interaction pairs predicted by the miRDB, miRTarBase, and TargetScan public databases. E) Venn diagram illustrating the intersection of these 1526 target mRNAs, the piRNA‐137463‐regulated DE mRNAs, and cholesterol metabolism‐related gene sets. F) The recruitment of LOC100128494 by AGO2 was detected using AGO2‐RIP assays in LUAD cells. G) miRanda software predicted the miR‐24‐3p binding site in LOC100128494 (LOC100128494‐WT2) and the designed mutant sequence (LOC100128494‐MUT2) is illustrated. H) The interaction between miR‐24‐3p and LOC100128494 was verified by dual luciferase reporter assays. I) miRanda software predicted the miR‐24‐3p binding site INSIG1 (INSIG1‐WT) and the designed mutant sequence (INSIG1‐MUT) is illustrated. J) The interaction between miR‐24‐3p and INSIG1 was verified by dual luciferase reporter assays. K) The effect of wild‐type or mutant LOC100128494 on INSIG1 protein expression in piRNA137463‐silenced cells treated with or without miR‐24‐3p mimics. Data are presented as mean ± SD. Statistical analyses were performed using t‐tests (F, H, and J) and one‐way ANOVA (B and K). ^*^ *P* < 0.05, ^**^ *P* < 0.01, ^***^ *P* < 0.001, *n.s*., not significant.

Existing research suggests that the expression of LOC100128494 is modulated by Epstein–Barr virus (EBV) in the context of EBV‐associated cancers.^[^ ^33^ ^]^ However, the potential role of LOC100128494 and its mechanism in LUAD have not been explored. The biological function of lncRNAs is closely linked to their subcellular localization. Prediction with lncLocator and iLoc‐LncRNA indicated that LOC100128494 is primarily expressed in the cytoplasm/cytosol (**Figure** **5**A). Cellular fractionation, RNA extraction, and RT‐qPCR analysis confirmed that LOC100128494 predominantly resides in the cytoplasm (Figure 5B). Consequently, we hypothesized that LOC100128494 may act as a ceRNA, sponging miRNAs to modulate the expression of downstream miRNA targets.^[^ ^34^ ^]^ miRcode algorithm predicted 61 downstream miRNAs that may interact with and be regulated by LOC100128494 (Figure 5C). Next, using miRDB, miRTarBase, and TargetScan databases, we identified a total of 5528 miRNA‒mRNA pairs involving 61 miRNAs potentially downstream of LOC100128494 and 1526 target mRNAs (Figure 5D and Table S1, Supporting Information). To identify which LOC100128494‐regulated mRNAs may be involved in piRNA‐137463‐mediated cholesterol metabolism, we took the intersection of the 1526 target mRNAs of LOC100128494, differentially expressed mRNAs by piRNA‐137463 inhibition, and cholesterol metabolism‐related gene (Figure 5E). We identified three genes at the intersection of these gene sets, including low‐density lipoprotein receptor (LDLR), fatty acid synthase (FASN), and INSIG1 (Figure 5E), which were involved in 14 miRNA‒mRNA molecular pairs (Table S2, Supporting Information). Reactome enrichment analysis revealed a potential involvement of piRNA‐137463 in the regulation of cholesterol biosynthesis through the SREBP pathway (Figure S3B, Supporting Information). Given that the expression of LOC100128494 is regulated by piRNA‐137463, we speculate that the INSIG1 gene, which is modulated by LOC100128494 through a ceRNA mechanism, may also be subject to regulation by piRNA‐137463. Consequently, we have formulated a hypothetical ceRNA network mediated by piRNA‐137463, involving LOC100128494/miR‐24‐3p/INSIG1.

To experimentally validate whether LOC100128494 functions as a ceRNA for miR‐24‐3p, we used Argonaute‐RNA immunoprecipitation (AGO2‐RIP) to detect the presence of ceRNA mechanisms involving LOC100128494. AGO2‐RIP and qPCR showed that LOC100128494 binds endogenously to AGO2, indicating that LOC100128494 could function as a ceRNA (Figure 5F). Using miRanda, we predicted the interaction sites between LOC100128494 and miR‐24‐3p (Figure 5G). The dual‐luciferase assay demonstrated that miR‐24‐3p induced a reduction in luciferase activity in the wild‐type LOC100128494‐WT2 construct, whereas this effect was not observed in the LOC100128494‐MUT2 construct, which harbors mutations at the predicted interaction sites (Figure 5H; Figure S4A, Supporting Information). Next, the miRanda algorithm was further employed to predict the binding sites of miR‐24‐3p in 3′ untranslated regions of INSIG1 mRNA (Figure 5I). Dual‐luciferase reporter assay revealed that mutation at the predicted interaction site failed to reduce the luciferase activity induced by the transfection of miR‐24‐3, suggesting that miR‐24‐3p exerts a direct inhibitory effect on INSIG1 mRNA (Figure 5J; Figure S4B, Supporting Information). Therefore, bioinformatic prediction and dual luciferase reporter assay confirmed the direct interaction between LOC100128494 and miR‐24‐3p, and miR‐24‐3p and INSIG1.

To further elucidate the regulatory relationship of gene expression among LOC100128494, miR‐24‐3p, and INSIG1, we assessed miR‐24‐3p and INSIG1 levels in cells with varying LOC100128494 expression. In LUAD cell lines, knockdown of LOC100128494 resulted in a significant upregulation of miR‐24‐3p expression, whereas INSIG1 expression was concomitantly reduced (Figure S4C–E, Supporting Information). Compared to the control vector, transfection with miR‐24‐3p mimics significantly reduced INSIG1 expression at both transcript and protein levels (Figure S4F,G, Supporting Information). Immunoblotting analyses further demonstrated that the overexpression of wild‐type LOC100128494 mitigated the inhibitory effect of miR‐24‐3p on INSIG1, whereas overexpression of the mutant LOC100128494 did not produce the same effect (Figure 5K). Taken together, these findings suggest that LOC100128494 directly binds to miR‐24‐3p, serving as a miR‐24‐3p sponge to suppress its regulatory impact on INSIG1 expression in LUAD cells.

### 2.7 piRNA‐137463 Controls the Expression of LOC100128494/miR‐24‐3p/INSIG1 Axis in LUAD Cells

Since piRNA‐137463 directly interacts with LOC100128494 and promotes its expression, it is highly likely that piRNA‐137463 further regulates the LOC100128494‐mediated ceRNA network (LOC100128494/miR‐24‐3p/INSIG1). As expected, Anta‐137463 effectively downregulated miR‐24‐3p expression while simultaneously upregulating INSIG1 mRNA and protein expression (Figure S4H–K, Supporting Information). Moreover, Anta‐137463 also inhibited mSREBP2 expression, which is a key transcription activator of genes involved in cholesterol synthesis and a downstream target of INSIG1 (Figure S4J,K, Supporting Information). Additionally, qPCR revealed that Anta‐137463 reduced the expression of genes downstream of mSREBP2 involved in *de novo* cholesterol synthesis (Figure S4L,M, Supporting Information). Therefore, these results suggest that piRNA‐137463 could modulate cellular cholesterol synthesis via the regulation of LOC100128494, miR‐24‐3p, INSIG1, and mSREBP2.

### 2.8 piRNA‐137463 Mediates the Malignant Phenotype of LUAD through Cholesterol Synthesis Mediated by LOC100128494 and INSIG

We proposed that the LOC100128494/miR‐24‐3p/INSIG1 ceRNA network is essential for the piRNA‐137463‐modulated malignant phenotypes observed above. CCK‐8 and EdU labeling assays demonstrated that siRNA‐mediated knockdown of LOC100128494 (siLOC) or INSIG1 (siIN) significantly reversed the inhibition of survival and proliferation caused by the attenuated piRNA‐137463 expression after Anta‐137463 treatment (Figure S5A–C, Supporting Information). Meanwhile, knocking down the expression of LOC100128494 or INSIG1 reversed the inhibitory effect of Anta‐137463 on the migratory and invasive properties of LUAD cells (Figure S5D–G, Supporting Information), accompanied by a reversal of the suppression of the EMT process caused by piRNA‐137463 silencing, which was verified by western blotting and immunofluorescence of EMT markers (Figure S6A–D, Supporting Information). Consistently, the knock‐down of LOC100128494 or INSIG1 also reversed the effect of piRNA‐137463 silencing on T cell‐mediated anti‐tumor immunity (Figure S7A–F, Supporting Information). Additionally, the knock‐down of LOC100128494 or INSIG1 also abolished Anta‐137463‐induced reduction in cholesterol levels and the expression of cholesterol synthesis‐related genes, namely INSIG1 and mSREBP2 (Figure S8A–D, Supporting Information), lipid raft levels (Figure S9A, Supporting Information), the expression of lipid raft markers (Figure S9B, Supporting Information), and PD‐L1 expression (Figure S10A,B, Supporting Information) in A549 and 95D cells. PD‐L1 ubiquitination in cells treated with Anta‐137463 was also reduced by the knock‐down of LOC100128494 or INSIG1 (Figure S10C, Supporting Information). Taken together, our data suggested that piRNA‐137463 promotes malignant‐phenotype progression, cholesterol synthesis, lipid raft content, and PD‐L1 expression in a LOC100128494/INSIG‐dependent manner.

### 2.9 Anta‐137463 Treatment Inhibits Tumor Growth and Metastasis via LOC100128494 in Nude Mice

**Figure 6:**
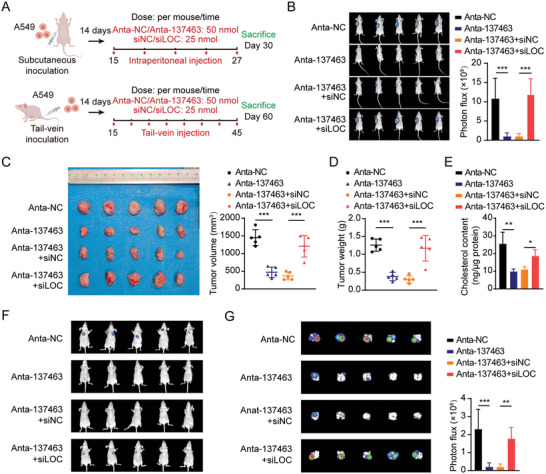
piRNA‐137463 promotes tumor growth and metastasis via LOC100128494 in nude mice. A) Diagram illustrating the construction and treatment protocols for subcutaneous xenograft tumor and metastatic tumor models in nude mice. Mice were divided into four experimental groups: Anta‐NC Anta‐137463, Anta‐137463+siNC and Anta‐137463+siLOC. B) Bioluminescence images (left panel) and quantification of bioluminescence intensities (right panel) of subcutaneous xenografts in mice from the indicated groups. C) Tumor volume in mice from the indicated groups. D) Tumor weight of nude mice in mice from the indicated groups. E) Cholesterol levels in tumor tissue of the xenografts in mice from the indicated groups. F) Bioluminescence images of tumors in mice from different treatment groups in lung metastasis models. G) Bioluminescence image (left panel) and bioluminescence intensity quantification (right panel) of metastases in the lungs from the indicated groups. Data are presented as mean ± SD. Statistical analysis was performed using one‐way ANOVA (B–E,G). ^*^ *P* < 0.05, ^**^ *P* < 0.01, ^***^ *P* < 0.001.

To investigate the oncogenic roles and mechanisms of piRNA‐137463 in vivo, athymic mice were randomly stratified into four cohorts: I) Anta‐NC, II) Anta‐137463, III) Anta‐137463+siNC, and IV) Anta‐137463+siLOC. The detailed procedures for using the four reagents in the subcutaneous xenograft tumor model and lung metastasis models are shown in **Figure** **6**A. On day 30, the tumor‐associated fluorescence intensity (Figure 6B), tumor volume (Figure 6C), and tumor weight (Figure 6D) in the Anta‐137463 group were reduced, accompanied by a decrease in cholesterol content (Figure 6E), compared with those in the Anta‐NC group. Immunohistochemistry (IHC) assays revealed higher levels of INSIG1 expression in Anta‐137463 group (II) compared to g Anta‐NC group (I) whereas the expression of SREBP2, FLOT1, and Ki67 was markedly diminished within the xenograft tumor tissues (Figure S11, Supporting Information). Compared with the Anta‐137463+siNC group (III), the Anta‐137463+siLOC group (IV) exhibited an increased tumor burden (Figure 6B–D) and higher expression of SREBP2, FLOT1, and Ki67, but lower INSIG1 expression (Figure S11, Supporting Information), suggesting that the effects of piRNA‐137463 depletion on tumor growth and cholesterol biosynthesis were dependent on LOC100128494.

In the lung metastasis models, administration of Anta‐137463 (group II) significantly reduced the metastatic potential of LUAD cells in tail vein‐injected nude mice, as evidenced by decreased luciferase signals both in vivo and *ex vivo*, and fewer lung metastatic nodules, as confirmed by hematoxylin and eosin (H&E) staining (Figure 6F,G; Figure S12A, Supporting Information), compared to the Anta‐NC group (I). Furthermore, immunohistochemical analysis of serial lung tissue sections from tail vein‐injected mice revealed elevated levels of INSIG1 and E‐cadherin, but lower SREBP2 and FLOT1 staining in lung metastasis tissues from Anta‐137463 group (II) compared to those from Anta‐NC group (I) (Figure S12B, Supporting Information). LOC100128494 silencing attenuated the effect of Anta‐137463 on tumor metastasis and cholesterol levels in the lung metastasis models using nude mice (Figure 6F,G; Figure S12B, Supporting Information). Therefore, our findings suggest that targeting piRNA‐137463 treatment may block tumor progression via LOC100128494 in LUAD.

### 2.10 Anta‐137463 Treatment Boosts the Response of LUAD to Anti‐PD‐1 Therapy in vivo

**Figure 7:**
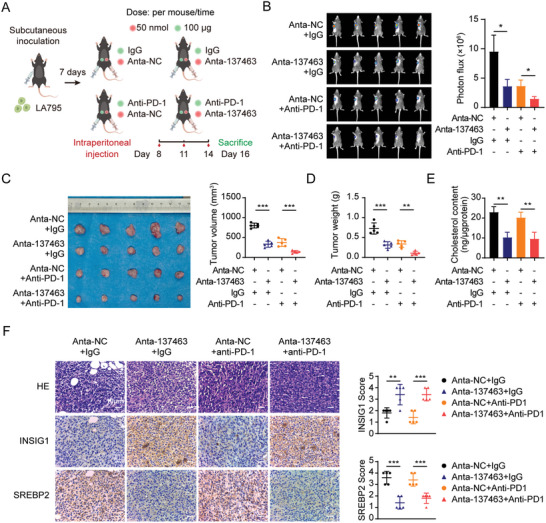
Anta‐137463 treatment enhances the response of LUAD to anti‐PD‐1 therapy in vivo. A) Diagram illustrating the construction and treatment protocol for a subcutaneous xenograft model in C57BL/6J mice. Mice were divided into four experimental groups: Anta‐NC + IgG, Anta‐137463 + IgG, Anta‐NC + Anti‐PD‐1, and Anta‐137463 + Anti‐PD‐1. The experiment was terminated at the indicated time points. B) Bioluminescence images (left panel) and quantification of bioluminescence intensities (right panel) of subcutaneous xenografts in mice from the indicated groups. C) Tumor volume in mice from the indicated groups. D) Tumor weight in mice from the indicated groups. E) Cholesterol levels in tumor tissue of xenografts in mice of the indicated groups. F) Representative images of H&E staining and IHC for INSIG1 and SREBP2 in xenograft sections (left panel) and statistical graphs of IHC scores (right panel). Data are presented as mean ± SD. Statistical analysis was performed using one‐way ANOVA (B‐F). ^*^ *P* < 0.05, ^**^ *P* < 0.01, ^***^ *P* < 0.001.

Lowering cholesterol levels has been shown to augment the efficacy of immune checkpoint therapy in cancer.^[^ ^35^ ^]^ We aimed to determine whether Anta‐137463 treatment could potentiate the responsiveness of tumors to immune checkpoint therapy. C57BL/6J murine models were inoculated with murine LUAD cell lines and subsequently administered with either PD‐1 monoclonal antibody (Anti‐PD‐1) or the corresponding IgG isotype control (Anti‐IgG), following the regimen shown in **Figure** **7**A. Anta‐137463 treatment reduced tumor‐associated fluorescence intensity (Figure 7B), tumor volume (Figure 7C), tumor weight (Figure 7D), and cholesterol content (Figure 7E), compared with those treated with Anta‐NC. The combined application of Anta‐137463 and Anti‐PD‐1 substantially reduced tumor mass, weight, volume, and cholesterol content compared to other groups, suggesting that the greatest decrease in oncogenic burden was achieved by the co‐application of Anta‐137463 and Anti‐PD‐1 (Figure 7B–E). Regardless of Anti‐IgG or Anti‐PD‐1 treatment, Anta‐137463 treatment increased the staining level of INSIG1 and reduced the level of SREBP2 in serial sections of xenograft tumor tissues (Figure 7F). In summary, our findings indicate that targeted inhibition of piRNA‐137463 in combination with anti‐PD‐1 therapy exhibits a synergistic antitumor effect in LUAD.

## 3 Discussion

**Figure 8:**
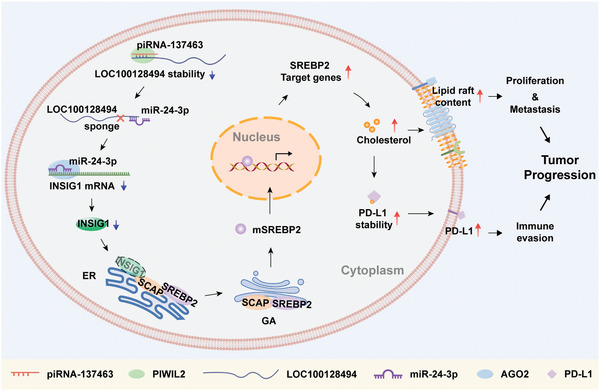
A schematic diagram of how piRNA‐137463 promotes LUAD progression. piRNA‐137463 interacts with PIWIL2 to form the PIWI–piRNA complex, which promotes the degradation of LOC100128494. As a ceRNA sponge, the downregulation of LOC100128494 decreases its interaction with miR‐24‐3p. The liberated miR‐24‐3p then binds to INSIG1, resulting in reduced INSIG1 expression. The decreased levels of INSIG1 fail to retain the SCAP‐SREBP2 complex in the ER leading to its translocation to the Golgi apparatus. This process increases the levels of active mSREBP2, subsequently enhancing cholesterol biosynthesis. On one side, elevated cholesterol levels augment lipid raft content, promoting the proliferation, EMT, and metastasis of LUAD cells. On the other side, the increased cholesterol inhibits the ubiquitin‐proteasome degradation of PD‐L1, leading to its upregulation, which in turn facilitates immune escape. Together, the enhanced metastasis, proliferation, and immune evasion drive the progression of LUAD.

In this study, we identified a novel carcinogenic piRNA, piRNA‐137463, as an indicator of poor survival in LUAD, promoting proliferation, metastasis, and immune escape of LUAD cells. piRNA‐137463 may hold significant potential for the diagnostic processes, prognostic assessments, and targeted therapeutic interventions for LUAD and metastatic LUAD. **Figure** **8** elucidates the mechanism through which piRNA‐137463 contributes to tumor progression and unfavorable prognosis. piRNA‐137463 forms PIWI–piRNA complex with PIWIL2, which subsequently facilitates the degradation of LOC100128494. As a ceRNA sponge, the downregulation of LOC100128494 releases miR‐24‐3p, allowing miR‐24‐3p to downregulate the expression of INSIG1 by binding to INSIG1 mRNA. Downregulated INSIG1 cannot retain the SCAP‐SREBP2 complex in the ER, resulting in its translocation to the Golgi apparatus, leading to an increased level of active mSREBP2 and subsequent upregulation of cholesterol biosynthesis‐related genes. On the one hand, the increased cholesterol promotes the content of lipid rafts, thereby enhancing the proliferation, EMT, and metastasis of LUAD cells. On the other hand, the increased cholesterol upregulates PD‐L1 by inhibiting the ubiquitin‐proteasome degradation, thereby facilitating immune escape. The enhanced metastasis, proliferation, and immune escape ultimately result in LUAD progression.

Cholesterol metabolism reprogramming in cancer cells encompasses four principal features: enhanced cholesterol biosynthesis and uptake, reduced efflux, and the accumulation of cholesterol derivatives.^[^ ^36^ ^]^ In our study, we propose that piRNA‐137463 promotes cholesterol biosynthesis, leading to enhanced tumor proliferation, EMT, metastasis, and immune evasion. Beyond these effects, increased cholesterol synthesis also contributes to resisting cell death, regulating cancer stem cells, facilitating angiogenesis, inducing DNA damage and genomic instability, fostering tumor‐promoting inflammation, and influencing the polymorphic microbiome.^[^ ^37^ ^]^ The specific mechanism by which piRNA‐137463 promotes tumor progression warrants further exploration in future studies. Targeting lipid metabolism, including cholesterol, is important for inhibiting tumorigenesis and overcoming treatment resistance.^[^ ^38^ ^]^ Multiple clinical studies suggest that statin, which targets cholesterol biosynthesis via 3‐hydroxy‐3‐methylglutaryl‐CoA reductase, in combination therapy enhances survival and improves outcomes in metastatic pancreatic cancer,^[^ ^39^ ^]^ breast cancer,^[^ ^40^ ^]^ lung cancer,^[^ ^41^ ^]^ and other malignancies.^[^ ^42^ ^]^ Additionally, other cholesterol‐targeting drugs, such as those targeting SREBPs (such as fatostatin) and ezetimibe, which act on NPC‐1L1, have shown promise in inhibiting tumor progression.^[^ ^43^ ^]^ These drugs hold significant potential as candidates for anti‐tumor therapies. Our research highlights an important role of piRNA‐137463 in cholesterol metabolism‐related mechanisms underlying tumor progression and may have significant implications for the precise selection of LUAD patients who would benefit from cholesterol‐targeting drug therapy in a clinical setting.

The discovery of ncRNAs such as miRNAs, lncRNAs, circular RNAs, and piRNAs, and their roles in post‐transcriptional gene regulation, has been a major focus of research over the past three decades.^[^ ^44^ ^]^ In this study, we identified that piRNA‐137463 facilitates the degradation of LOC100128494 via complementary base pairing, thereby regulating the ceRNA axis of LOC100128494/miR‐24‐3p/INSIG1. Consistent with our findings, previous studies have highlighted the involvement of piRNAs in ceRNA networks.^[^ ^7^, ^45^ ^]^ For instance, piR‐36712 regulates the SEPW1 pseudogene (SEPW1P)/miR‐7/SEPW1 ceRNA networks by silencing SEPW1P, thereby inhibiting the expression of the p53 and mitigating breast cancer progression and chemoresistance.^[^ ^7b^ ^]^ Liu et al. reported that piR‐30188‐PIWIL3 inhibits the progression of neurogliomas through the OIP5‐AS1/miR‐367‐3p/CEBPA ceRNA axis.^[^ ^45^ ^]^ Cytoplasmic piRNAs can bind to proteins and regulate the translation and expression of target genes.^[^ ^9^ ^]^ Future research could explore whether piRNA‐137463 interacts with proteins, identify the protein targets, and elucidate its functional role in protein regulation.

Our findings indicated that piRNA‐137463 enhances both immune evasion and insensitivity to PD‐1 inhibitors, which can be explained by the role of piRNA‐137463 in promoting cholesterol biosynthesis. Cholesterol can promote immune evasion through multiple mechanisms.^[^ ^36a^ ^]^ Cholesterol within the tumor microenvironment promotes the exhaustion of CD8^+^ T cells via an ER stress‐dependent pathway involving X‐box binding protein 1 (XBP1), resulting in the upregulation of programmed cell death protein 1 (PD‐1) and other immune checkpoint molecules on T cells.^[^ ^46^ ^]^ XBP1 can also augment immunosuppression by activating bone marrow‐derived suppressor cells (MDSC).^[^ ^47^ ^]^ Cholesterol accumulation damages T cell receptor (TCR) dimer formation, disrupts TCR signal transduction and inhibits T cell activation.^[^ ^48^ ^]^ Cholesterol accumulation triggers the phosphorylation of activated linker for T cell activation and facilitates the release of proprotein convertase subtilisin/kexin type 9 (PCSK9). PCSK9 subsequently binds to LDLR, obstructing the recycling of both LDLR and TCR to the plasma membrane. This disruption in recycling leads to a diminished effector function of cytotoxic T‐lymphocytes.^[^ ^49^ ^]^ Oxysterols secreted by cancer cells inhibit dendritic cells' ability to present tumor antigens to T cells by reducing the expression of C‐C chemokine receptor type 7.^[^ ^50^ ^]^ Wang et al. reported that cholesterol directly binds to PD‐L1 protein and enhances its expression in colon and leukemia cell lines.^[^ ^30^ ^]^ Our study consistently verified that piRNA‐137463 can enhance the PD‐L1 expression in LUAD cells by decreasing its ubiquitination‐proteasome degradation and increasing its stability. It is still worth exploring in the future whether piRNA‐137463 induces immune evasion through other cholesterol‐mediated mechanisms such as antitumor immune suppression, inhibitory tumor immune microenvironment (such as the exhaustion of CD8^+^ T cells), which could enhance the efficacy of PD‐1 inhibitors.

## 4 Conclusion

In conclusion, our findings identify piRNA‐137463 as a novel diagnostic and prognostic biomarker that modulates LUAD proliferation, metastasis, and immune escape. Furthermore, we provide the first evidence of piRNA‐137463‐mediated regulation of the LOC100128494/miR‐24‐3p/INSIG1 axis, which controls cholesterol biosynthesis and contributes to LUAD progression. These results highlight cholesterol increase induced by piRNA‐137463 as a key mechanism of tumor progression, offering valuable insights and potential strategies for clinical intervention in LUAD, including early diagnosis, precision therapy, novel therapeutic targets, and enhanced immunotherapy efficacy.

## 5 Experimental Section

### Public Data Mining and RNA–RNA Interaction Prediction

Gene annotation information for human‐specific piRNAs was obtained from piRBase.^[^ ^26^ ^]^ piRNA transcriptome data from LUAD and normal lung tissues were accessed using the piRNA‐eQTL database,^[^ ^51^ ^]^ with clinical information retrieved from the TCGA database. Potential RNA targets of piRNAs were predicted using the PITA^[^ ^52^ ^]^ and miRanda^[^ ^53^ ^]^ programs. Downstream miRNAs potentially interacting with and regulated by lncRNAs were identified using the miRcode algorithm.^[^ ^54^ ^]^ miRNA‒mRNA pairs were obtained using three publicly available algorithms: miRDB,^[^ ^55^ ^]^ miRTarBase,^[^ ^56^ ^]^ and TargetScan.^[^ ^57^ ^]^

### Cell Culture and Reagents

Human LUAD cell lines (A549, 95D, PC9, H827, H1299, and H1650), human Jurkat T cells, and the murine LA795 cells were maintained in RPMI 1640 medium (Gibco) containing 10% (v/v) fetal bovine serum (164210, Procell) and 1% penicillin‐streptomycin (SV30010, HyClone) at 37 °C with 5% CO_2_. An 8‐h treatment with 10 µm MG132 (HY‐13259, MedChemExpress) or 10 mm NH_4_Cl (12125‐02‐9, Aladdin) was used for the protein degradation assays. Cycloheximide (10 µg mL^−1^, HY‐1232010, MCE) was added to the culture media for investigation of protein stability.

### Reverse Transcription (RT) at Low dNTP Concentration Followed by PCR (RTL‐P)

RTL‐P assays in A549 and 95D cells were performed according to previously described methods.^[^ ^58^ ^]^ Cellular RNA was extracted using Trizol Reagent (15596026, Thermo Fisher) and ligated to 3′ RNA junctions (Severn) using T4 RNA ligase (2050A, Takara). The ligated products underwent reverse transcription into complementary DNA using either high (40 µm) or low (0.4 µm) dNTP concentrations, with or without anchoring RT primers for modified nucleotides. PCR amplification of the products was performed using specific primers (Table S3, Supporting Information) and Ex Taq polymerase (RR001A, Takara), with pre‐denaturation at 94 °C for 1 min, followed by 40 cycles of annealing at 94 °C for 20 s and elongation at 60 °C for 20 s. PCR products were analyzed using agarose gel electrophoresis.

### Western Blotting

Standard Western blotting was performed following previously described protocols.^[^ ^59^ ^]^ The antibodies used in the study are listed in Table S4 (Supporting Information). Protein levels were quantified relative to β‐actin or GAPDH in the same sample and normalized against the corresponding control.

### RNA Isolation and qPCR Analysis

Total RNA was extracted using TRIzol after harvesting cells by incubating them with 0.25% (v/v) trypsin at 37 °C for 1–3 min. RNA from nuclear and cytoplasmic fractions was extracted using the PARIS kit (AM1921, Thermo Fisher) according to the manufacturer's instructions. For qPCR, total RNA was reverse transcribed into cDNA using the Transcriptor First Strand cDNA Synthesis Kit (04897030001, Roche) with either random primers or specific miRNA/piRNA stem‐loop reverse transcription primers, following the manufacturer's protocol.

The relative expression of piRNA‐137463 was quantified using a specific Hairpin‐itTM Real‐Time PCR kit (E2204, GenePharma) on the StepOne Plus quantitative PCR system (Applied Biosystems). The expression levels of lncRNAs, miRNAs, and mRNAs were quantified using primers listed in Table S3 (Supporting Information) and the Talent qPCR Premix (FP209‐02, TIANGEN). The qPCR reaction conditions were as follows: pre‐denaturation at 95 °C for 3 min, followed by 40 cycles of denaturation at 95 °C for 5 s, and extension at 60 °C for 15 s. GAPDH was used as the endogenous control for mRNAs and lncRNAs, while U6 was used for miRNAs and piRNAs. Relative quantification was performed using the ΔΔCq method, with normalization to endogenous controls and control samples.

### Biotinylated RNA Pull‐Down Assay

Small RNA‐interacting molecules were identified using the miRNA pull‐down kit (Bes5108, BersinBio). Briefly, A549 and 95D cells were transfected with 100 nm biotinylated piRNA‐137463 probes (GenePharma) or negative control probes, using the jetPRIME Transfection Reagent (Polyplus). After 48 h, cells were lysed and incubated with pre‐blocked magnetic beads at 4 °C for 4 h. Interacting molecules were then eluted using RNA or protein elution buffer. The abundance of piRNA‐137463 interacting molecules in the sample was analyzed by Western blotting or qPCR.

### Antagomir and Agomir Treatment for piRNA‐137463 Expression Regulation

Chemically modified *vivo*‐grade antagopiR‐137463 or agomir‐137463, specific to piRNA‐137463, were synthesized by GenePharma. Non‐targeting antagoNC was used as a negative control. Cells were treated with 50 nm antagomir‐137463, agomir‐137463, or the corresponding negative control using GP‐transfect‐Mate (G04008, GenePharma) following the manufacturer's protocol. After 24 h, cells were harvested for subsequent analyses. The sequences of antagopiR‐137463 and agomir‐137463 are listed in Table S5 (Supporting Information).

### siRNA Transfection

Cells were seeded at 2 × 10^3^ cells cm^−2^ and 24 h later, transfected with 20 nm siRNAs (GenePharma; Table S5, Supporting Information) or plasmids using the jetPRIME. After 6 h of incubation, the transfection mixture was replaced with fresh complete media, and the cells were cultured for an additional 24–48 h before analysis.

### RNA‐Seq and Functional Enrichment Analyses

Total RNA extracted using TRIzol was used for bulk RNA‐seq transcriptome library preparation with the Illumina Stranded mRNA Prep, Ligation Kit (Illumina, USA). Paired‐end 2 × 150 bp next‐generation sequencing was performed using the Illumina NovaSeq 6000 (Illumina). Raw sequencing data were aligned to the *Homo sapiens* GRCh38.p13 genome using HISAT2.^[^ ^60^ ^]^ Gene counts were quantified and normalized using the RSEM^[^ ^61^ ^]^ method in R, and differential expression analysis was performed with DESeq2^[^ ^62^ ^]^ in R, comparing Anta‐137463 with Anta‐NC groups, with significance thresholds of |FC| > 1.2 and P value < 0.05. Over‐representation analysis was performed using the Majorbio Cloud Platform (Majorbio), including GO term overrepresentation analysis^[^ ^63^ ^]^ with Fisher Exact test followed by BH correction, KEGG analysis,^[^ ^64^ ^]^ and Reactome analysis.^[^ ^65^ ^]^

### RNA Stability Assay

To determine the half‐life of lncRNAs, A549 and 95D cells were cultured in complete media with 2 mg mL^−1^ actinomycin D (HY‐17559, MCE) or an equivalent volume of Dimethyl sulfoxide. Total RNA was extracted at 0, 3, and 6 h. The abundance of lncRNAs was quantified using qPCR.

### FISH

The piRNA‐137463 probe conjugated with Cy3 was synthesized by GenePharma. FISH was conducted using the RNA FISH kit protocol (F11101/50, GenePharma). A549 and 95D cells were fixed with 4% paraformaldehyde for 15 min at room temperature, treated with 0.1% (v/v) Triton X‐100 in Phosphate Buffered Saline (PBS) for 15 min, and incubated with 2 × SSC for 30 min at 37 °C. The cells were then hybridized with the piRNA‐137463 probe overnight at 37 °C, protected from light, and on the next day, incubated with 4′,6‐diamidino‐2‐phenylindole (DAPI; AR1176, BOSTER) at room temperature for 10 min. Samples were imaged using fluorescence confocal microscopy (Olympus, Japan).

### Argonaute 2‐RNA Immunoprecipitation (AGO2‐RIP) Assay

AGO2‐RIP experiments were performed using the RNA Immunoprecipitation Kit protocol (Bes5101, BersinBio). Briefly, A549 and 95D cells were transfected with miR‐24 mimic 24 h before cell lysis on ice. Lysed samples were incubated with anti‐AGO2 antibody or IgG (Table S4, Supporting Information) at 4 °C for 16 h. The precipitates were collected by the addition of well‐equilibrated magnetic beads, followed by 10 washes with polysome lysis buffer. RNAs in the precipitates were extracted using TRIzol and quantified by qPCR.

### Ubiquitination Assay

The A549 and 95D cells were transfected with HA‐Ub plasmid for 48 h and incubated with 10 µm MG132 for 8 h. Immunoprecipitation was conducted as previously described.^[^ ^59^ ^]^ The ubiquitination of PD‐L1 was measured by immunoblotting with an anti‐HA antibody (Table S4, Supporting Information).

### Cell Proliferation and Viability Assays

A549 and 95D cells were used for cell proliferation and viability assays. For the CCK‐8 proliferation assays, 2000 cells were seeded per well in a 96‐well plate and allowed to proliferate for 24, 48, or 72 h. After each time point, cells were incubated for 2 h with the CCK‐8 reagent (CK04, Dojindo). Absorbance was then quantified at 450 nm using a microplate reader (ELx800, BioTek). Cell proliferation was also assessed by EdU incorporation using the BeyoClick EdU‐555 Cell Proliferation Assay Kit (C0075S, Beyotime), according to the manufacturer's protocol. Cells were exposed to a 10 µm EdU for 2 h before fluorescence images were captured using fluorescence microscopy. For colony formation assays, 500 cells were seeded into each well of a 6‐well plate and cultured in growth media for 14 days. Afterward, colonies were fixed with 4% paraformaldehyde, stained with 0.5% crystal violet, and visualized using an inverted microscope (ECLIPSE Ts2, Nikon)

### Cell Migration and Invasion Assays

To assess the invasive and migratory ability of A549 and 95D cells, cells were pre‐incubated in serum‐free medium for 12 h and transwell chambers (3422, Corning) were utilized, with 4 × 10^4^ placed in the upper chamber in 200 µL of serum‐free medium. A Matrigel‐coated membrane was used for the invasion assays, but not for the migration assays. The lower chamber was supplemented with 600 µL of medium containing 20% (v/v) fetal bovine serum. Cells were incubated at 37 °C for 24 h for migration assays or 48 h for invasion assays. After incubation, the cells that had traversed the membrane were fixed with 4% paraformaldehyde, stained with 0.5% crystal violet, and visualized using an inverted microscope (ECLIPSE Ts2, Nikon).

### Wound Healing Assay

In the wound healing assays, a vertical cross wound was created on a confluent monolayer of A549 or 95D cells using a sterile 10‐µL pipette tip. The plate was washed with PBS, and then 2 mL of fresh serum‐free medium was added. The cells were stained with 0.5% crystal violet, and photographs were taken at 0 and 48 h after scratching.

### Plasmid Construction and Dual‐Luciferase Reporter Assays

Wild‐type plasmids (LOC100128494‐WT1, LOC100128494‐WT2, INSIG1‐WT) were created by inserting full‐length LOC100128494 or INSIG1 cDNAs into the XhoI and NotI sites of PHY‐811 reporter vectors. Mutant plasmids (LOC100128494‐MUT1) were constructed by introducing mutations at the predicted binding sites for piRNA‐137463 and LOC100128494. Similarly, mutant plasmids (LOC100128494‐MUT2) were created by targeting the predicted binding sites for LOC100128494 and miR‐24‐3p. Additionally, mutant plasmids (INSIG1‐MUT) were developed with mutations at the predicted binding sites for miR‐24‐3p and INSIG1. All synthesized vectors were obtained from Hanyin Biotechnology. For luciferase assays, 2000 A549 or 95D cells were seeded per well in 96‐well plates and transfected with 5 µg of plasmid and 100 pmol of agomiR‐137463 or miR‐24 mimic (GenePharma) using jetPRIME. After 48 h, cells were lysed using 1 × PLB according to the recommended protocol of the Dual‐Luciferase Assay System (E1910, Promega). Firefly luciferase activity was measured using LAR II, and Renilla luciferase activity was assessed with Stop & Glo, both on the SpectraMax i3x Multi‐Mode Detection Platform (Molecular Device).

### Immunofluorescence

A549 or 95D cells at a density of 2 × 10^4^ cells cm^−2^ were distributed into confocal dishes (801001, Nest) and, following a 24‐h incubation period, were fixed with 4% paraformaldehyde for 15 min at 4 °C. After fixation, cells were permeabilized using 0.1% (v/v) Triton X‐100 in PBS for 10 min and then blocked with goat serum (AR0009, Boster) for an additional 30 min at room temperature. Thereafter, cells were incubated with primary antibodies diluted in a 5% bovine serum albumin solution (AR0004, Boster) at 4 °C overnight. On the following day, cells were incubated with fluorescence‐conjugated secondary antibodies, also diluted in 5% bovine serum albumin solution in the dark at room temperature for 1 h, followed by counterstaining with DAPI. Finally, samples were examined using a fluorescence microscope (DMi8, Leica). Antibodies used in this study are listed in Table S4 (Supporting Information).

### Cholesterol Content Detection Assay

Following the protocol of the Tissue and Cell Total Cholesterol Content Enzymatic Assay Kit (E1015, Applygen), 1 × 10^6^ A549 or 95D cells or 10 mg of tissue were collected in 100 µL of lysate, shaken or homogenized, and allowed to settle for 10 min. The protein concentration was measured using the BCA method. The lysate supernatant was then heated at 70 °C for 10 min and centrifuged at 2000 × g for 5 min at room temperature. The supernatant was collected for testing. To prepare the assay, R1 and R2 reagents were mixed in a 4:1 ratio to create the working solution, and the cholesterol standard was diluted with anhydrous ethanol to create different concentration gradients. A total of 190 µL of the working solution was added to each well in a 96‐well plate, and cholesterol standard and samples to be tested were added separately. The reaction was incubated at 37 °C for 20 min, and the optical density (OD) value was measured at 570 nm. A standard curve was plotted to calculate the cholesterol concentration. Cholesterol content was normalized to per µg of protein.

### Filipin III Staining Assay

Filipin III (MB1848, Dalian Meilun Biotech) was used to label free cholesterol in A549 and 95D cells.^[^ ^28^ ^]^ A total of 1.5 × 10^5^ cells were cultured in confocal dishes for 24 h and fixed with 4% paraformaldehyde at room temperature for 30 min. 0.05 mg mL^−1^ Filipin III in PBS was added to the cells and incubated at room temperature for 1 h, shielded from light. This was followed by a 30‐min incubation with SYTOX Deep Red (S11380, Thermo Fisher) to stain the nucleus. Samples were imaged using fluorescence microscopy.

### Lipid Raft Labeling Assay

The Vybrant Alexa Fluor 488 Lipid Raft Labeling Kit (V‐34403, Thermo Fisher) protocol was followed for the lipid raft labeling process. A549 and 95D cells were labeled with CT‐B conjugated to Alexa Fluor 488 for 10 min, followed by a 15‐min cross‐linking reaction with an anti‐CT‐B antibody at 4 °C. The cells were subsequently fixed with 4% paraformaldehyde for 15 min and permeabilized with 0.1% (v/v) Triton X‐100 in PBS for 10 min at 4 °C. Nuclear staining was performed with DAPI, and the cells were observed using fluorescence microscopy.

### T Cell‐Mediated Tumor Cell Killing Experiments

For the activation of Jurkat T cells, the ImmunoCult Human CD3/CD28/CD2 T Cell Activator (10970, STEMCELL Technologies) was applied. The activated T cells were co‐cultured with A549 or 95D cells in 6‐well plates for 48 h, maintaining an effector‐to‐target (E/T) cell ratio of 5:1. After the co‐culture period, the supernatants were collected to assess the concentration of IFN‐γ secreted by the T cells using a human IFN‐γ ELISA Kit (EH008, ExCell Bio), following the manufacturer's guidelines. The remaining viable cells in the 6‐well plates were stained with 0.5% crystal violet, and the OD values were determined at 570 nm with a microplate reader (ELx800, BioTek) to measure cell survival. Additionally, a 5:1 E/T culture system was set up in 96‐well plates for 48 h to measure LDH release from the killed tumor cells. T‐cell cytotoxicity was evaluated using the Lactate Dehydrogenase Cytotoxicity Assay Kit (C0016, Beyotime), following the manufacturer's protocol. The cytotoxic killing efficiency of T cells was calculated using OD values by the following formula:

(1) $$\mathrm{Cytotoxicity}\;\mathrm{rate}\;\left(\%\right)=\frac{\mathrm{coculture}-\mathrm{effector}\;\mathrm{alone}}{\mathrm{positive}\;\mathrm{control}-\;\mathrm{negative}\;\mathrm{control}}\times 100\%$$

In this formula, Coculture refers to the OD value of the group where T cells and tumor cells were cultured together; Effector alone is the OD value of the T cells alone; Positive control represents the maximum killing OD value of the tumor cells; and Negative control is the spontaneous OD value of the tumor cells without any coculture with T cells.

### Flow Cytometry Analysis

T cells were labeled with 1 µm CFSE (21888, Sigma–Aldrich) dissolved in PBS, and incubated at 37 °C for 15 min, after which complete media was added to stop the staining. A549 or 95D cells were co‐cultured with the labeled T cells and collected as cell suspensions. Apoptosis was assayed by suspending cells in a pre‐formulated 1 × binding buffer at a concentration of ≈1 × 10^6^ cells mL^−1^, followed by staining with the Annexin V‐FITC/PI Apoptosis Detection Kit (product code FXP018, 4A BIOTECH). After a 15‐min incubation, the cells were washed three times with PBS and subsequently subjected to analysis using a FACSCalibur instrument (BD Biosciences). Subsequent data processing and analysis were performed using FlowJo software.

### Animal Experiments

All animal studies were approved by the Ethics Committee of Harbin Medical University (Ethics Approval Number: KY2023‐74) and conducted in accordance with relevant institutional guidelines and regulations. The mice were housed in a temperature‐controlled, pathogen‐free room with a 12‐h light–dark cycle. For the antitumor investigation, drug administration was performed via intraperitoneal injection in the in vivo tumor growth and C57BL/6J murine models. In the in vivo tumor metastasis model, intravenous injection was administered through the tail vein.

To construct subcutaneous xenograft tumor models, 20 five‐week‐old female Balb/c nude mice were selected and divided into the following groups: I) Anta‐NC (Anta‐NC treatment), II) Anta‐137463 (Anta‐137463 treatment), III) Anta‐137463 + siNC (Anta‐137463 and siRNA‐negative control treatment), and IV) Anta‐137463 + siLOC (Anta‐137463 and siRNA‐LOC100128494). Each group consisted of 5 mice. Mice were injected subcutaneously in the axilla with 1.5 × 10^7^ luciferase‐labeled tumor cells. 14 days after injection, the mice were administered the corresponding drugs intraperitoneally every 3 days, for a total of 5 treatments. The dosage for Anta‐NC, Anta‐137463, and *vivo*‐siNC/siLOC (Ribobio) was 50 and 25 nmol per mouse per treatment, respectively. The experiments were terminated 30 days after tumor cell injection. Tumor size and weight were measured weekly, and tumor volume was calculated according to the formula: Tumor volume = 0.5 × length × width^2^.

For the metastatic tumor model, the groupings were the same as those used for the subcutaneous xenograft tumor models. Mice were injected with 1 × 10⁶ luciferase‐labeled tumor cells via the tail vein, and 14 days later, the corresponding drugs were administered via the tail vein twice a week for 4 weeks. Anta‐NC and Anta‐137463 were administered at 50 nmol per mouse per treatment, while *vivo*‐siNC and siLOC were administered at 25 nmol per mouse per treatment. The experiments were terminated 60 days after tumor cell injection.

To determine the effect of piRNA‐137463 on immunotherapy, 20 five‐week‐old female C57BL/6J mice were used to construct subcutaneous xenograft tumor models. The mice were divided into four groups: Anta‐NC + IgG, Anta‐137463 + IgG, Anta‐137463 + Anti‐PD‐1, and Anta‐137463 + Anti‐PD‐1, with 5 mice in each group. Mice were injected subcutaneously in the axilla with 3 × 10^6^ luciferase‐labeled tumor cells. Seven days after injection, the mice were administered the corresponding drugs intraperitoneally. Anta‐NC and Anta‐137463 were administered at a dosage of 50 nmol per mouse per treatment, while anti‐mouse PD‐1 antibody (RMP1‐14, MCE) or *InVivo* MAb IgG2a isotype control (BE0089, BioXcell) was administered at 100 µg per mouse per treatment on days 8, 11, and 14. Tumor size and weight were measured every 2 days. At the end of the experiment, the mice were imaged using the IVIS Illumina System (PerkinElmer) and euthanized. Xenograft tumors and lung metastatic nodules were harvested for subsequent analysis.

### Immunohistochemistry

Tissue sections were deparaffinized in xylene and subsequently rehydrated through a graded series of ethanol concentrations. To inactivate endogenous peroxidase, sections were incubated in 3% hydrogen peroxide. Antigen retrieval was performed by immersing the sections in Tris‐EDTA (pH 9.0) solution, followed by heating in a pressure cooker. After cooling, the sections were blocked with goat serum (Boster) for 1 h and then incubated with the primary antibody, diluted in 1% (v/v) BSA in PBS, at 4 °C overnight. The next day, secondary antibodies were applied and incubated for 30 min at room temperature. Protein visualization was achieved using DAB reagent, and nuclear counterstaining was performed with hematoxylin. Imaging of the sections was conducted using an inverted microscope (DM750, Leica). The antibodies used in this study are listed in Table S4 (Supporting Information).

### Statistical Analysis

All experiments were performed in triplicates. Statistical analyses were performed using GraphPad Prism 8.3.0. Student's t‐tests were employed for comparisons between two groups, and one‐way or two‐way ANOVA was utilized for comparisons among multiple groups. A *P*‐value of less than 0.05 was considered statistically significant.

### Ethics

The study was carried out in accordance with the principles of the declaration of Helsinki and was approved by the Ethics Committee of Harbin Medical University Cancer Hospital (Grant No. KY2023‐74). The patients/participants provided their written informed consent to participate in this study.

## Conflict of Interest

The authors declare no conflict of interest.

## Author Contributions

Y.Z. did conceptualization, investigation, methodology, and wrote the original draft. F.T. performed data curation, formal analysis, funding acquisition. W.F. did resources, software, did review & editing. X.L. did validation, reviewed & editing. X.W. did methodology. H.Z. did visualization. X.H. did visualization. X.W. did visualization. L.C. did supervision, funding acquisition, did review & editing. Y.S. performed supervision, funding acquisition, did review & editing. Y.X. performed conceptualization, funding acquisition, and did review & editing. All authors read and approved the final manuscript.

## Supporting information



Supporting Information

Supplemental Table 1

Supplemental Table 2

Supplemental Table 3

Supplemental Table 4

Supplemental Table 5

## Data Availability

The data that support the findings of this study are available from the corresponding author upon reasonable request.
