# Supplementary material for: Targeting piRNA‐137463 Inhibits Tumor Progression and Boosts Sensitivity to Immune Checkpoint Blockade via De Novo Cholesterol Biosynthesis in Lung Adenocarcinoma
Source: Adv Sci (Weinh). 2024 Dec 18;12(6):2414100. doi: 10.1002/advs.202414100 (PMC11809383; doi:10.1002/advs.202414100)
Supplement: Supplementary file 1 — Supporting Information [file ADVS-12-2414100-s002.docx]

Supporting Information

Targeting piRNA-137463 Inhibits Tumor Progression and Boosts Sensitivity to Immune Checkpoint Blockade via De Novo Cholesterol Biosynthesis in Lung Adenocarcinoma

Yuning Zhan^1,2^, Fanglin Tian^1^, Weina Fan^1^, Xin Li^1^, Xiangyu Wang^1^, Hongxia Zhang^4^, Xin Hong^1^, Xin Wang^1^, Li Cai^1,2*^, Yang Song^3*^, Ying Xing^1*^

Y. Zhan, F. Tian, W. Fan, X. Li, X. Wang, X. Hong, X. Wang, L. Cai, Y. Xing

The Fourth Department of Medical Oncology, Harbin Medical University Cancer Hospital, 150 Haping Road, Harbin, 150081, China
E-mail: caili@ems.hrbmu.edu.cn; xingying@hrbmu.edu.cn

Y. Zhan, L. Cai

NHC and CAMS Key Laboratory of Molecular Probe and Targeted Theranostics, Harbin Medical University, Harbin, 150001, China

Y. Song

The Department of Orthopedics, The Second Affiliated Hospital of Harbin Medical University, Harbin, 150001, China
E-mail: songyang@hrbmu.edu.cn

H. Zhang

Imaging Center, Harbin Medical University Cancer Hospital, Harbin, 150081, China

**This file includes:**

Figure S1 to S12

**Other Supplementary Information for this manuscript include the following:**

Tables S1 to S5

**Supplementary Figures**


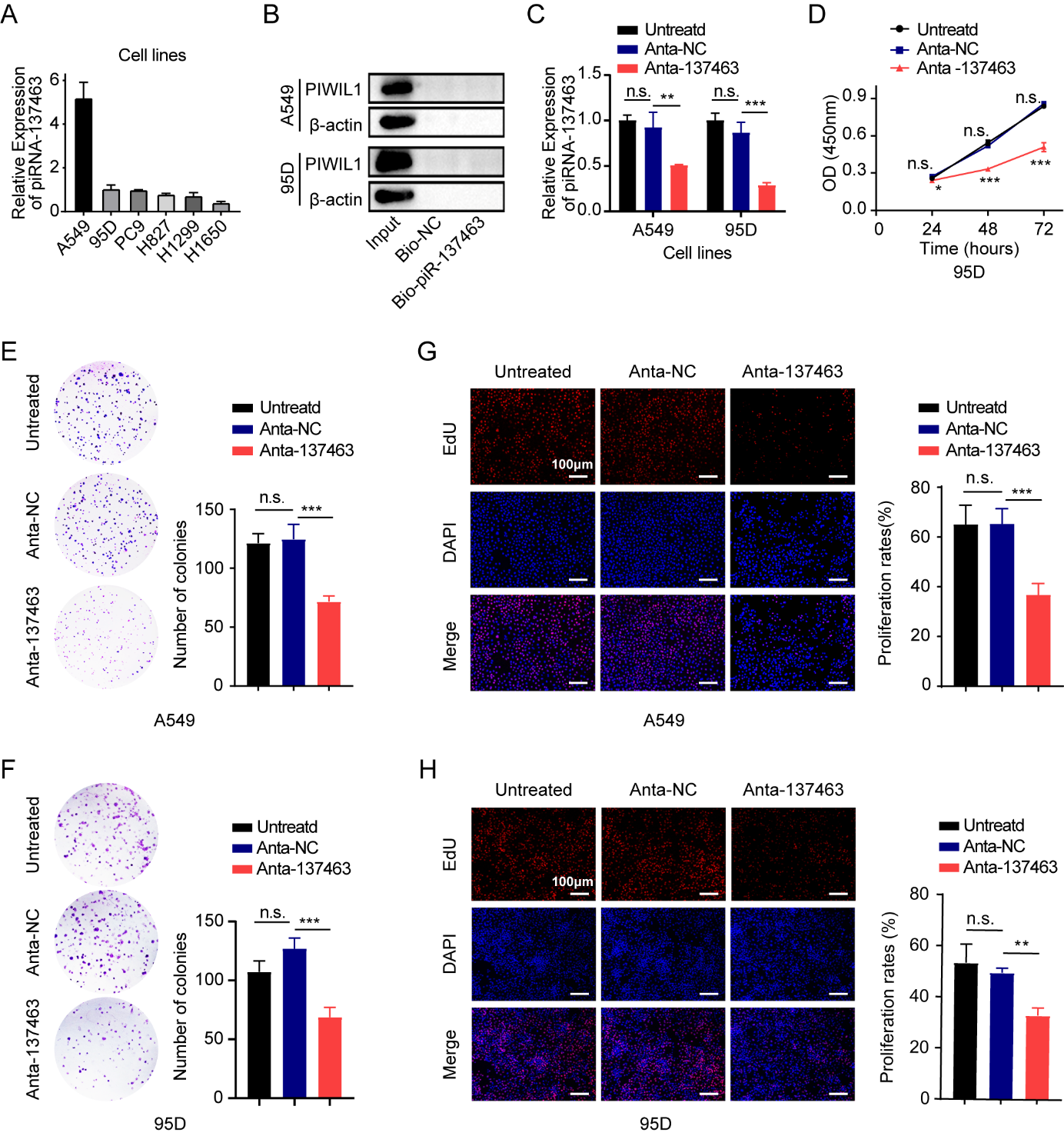


**Figure S1. Inhibition of piRNA-137463 impairs the proliferative capacity of LUAD cells.** A) Detection of piRNA-137463 expression in various LUAD cell lines by qPCR. B) Biotin-labeled piRNA pull-down assays to detect the binding potential of piRNA-137463 and PIWIL1 protein. C) qPCR results demonstrating that antagopiR-137463 effectively inhibited the expression of piRNA-137463 in A549 and 95D cells. D) Reduced proliferative capacity of 95D cells following piRNA-137463 silencing, analyzed by CCK-8 assays. E-H) Reduced proliferative capacity of LUAD cells following piRNA-137463 knockdown, analyzed by colony formation assays (E-F) and EdU incorporation assays (G-H). Data are presented as mean ± SD. Statistical analyses were performed using one-way ANOVA for (C, E-H) and two-way ANOVA for (D). **P* < 0.05, ***P* < 0.01, ****P* < 0.001, *n.s.*, not significant.

.


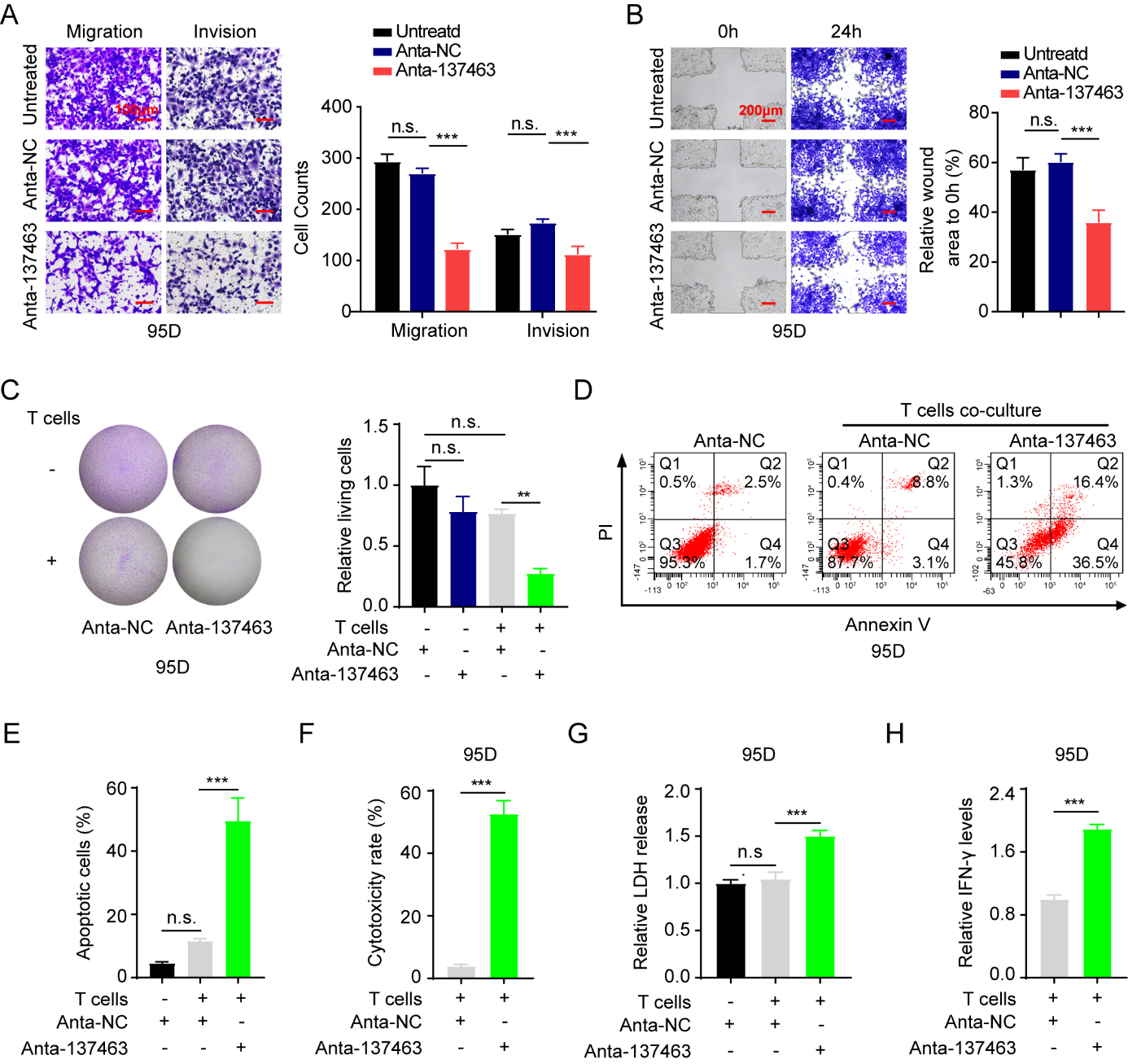


**Figure S2. Inhibition of piRNA-137463 impairs the malignant biological behavior of 95D cells.** A, B) The effects of piRNA-137463 inhibition on the invasive and migratory abilities of 95D cells were analyzed by (A) transwell assays and (B) wound healing assays. C) Crystal violet-stained images (left) and quantification (right) of 95D cells co-cultured with activated T cells in Anta-NC and Anta-137463. D, E) T cell-mediated apoptosis of 95D cells detected by flow cytometry in Anta-NC and Anta-137463. F) Effect of piRNA-137463 inhibition on the efficiency of T cell-mediated killing of 95D cells. G) Levels of LDH released from 95D cells co-cultured with T cells in Anta-NC and Anta-137463. H) Levels of IFN-γ secreted by T cells co-cultured with 95D cells in Anta-NC and Anta-137463. Data plotted as mean ± SD. Statistical analyses were performed using one-way ANOVA (A-C, E-H). ***P* < 0.01, ****P* < 0.001, *n.s.*, not significant.


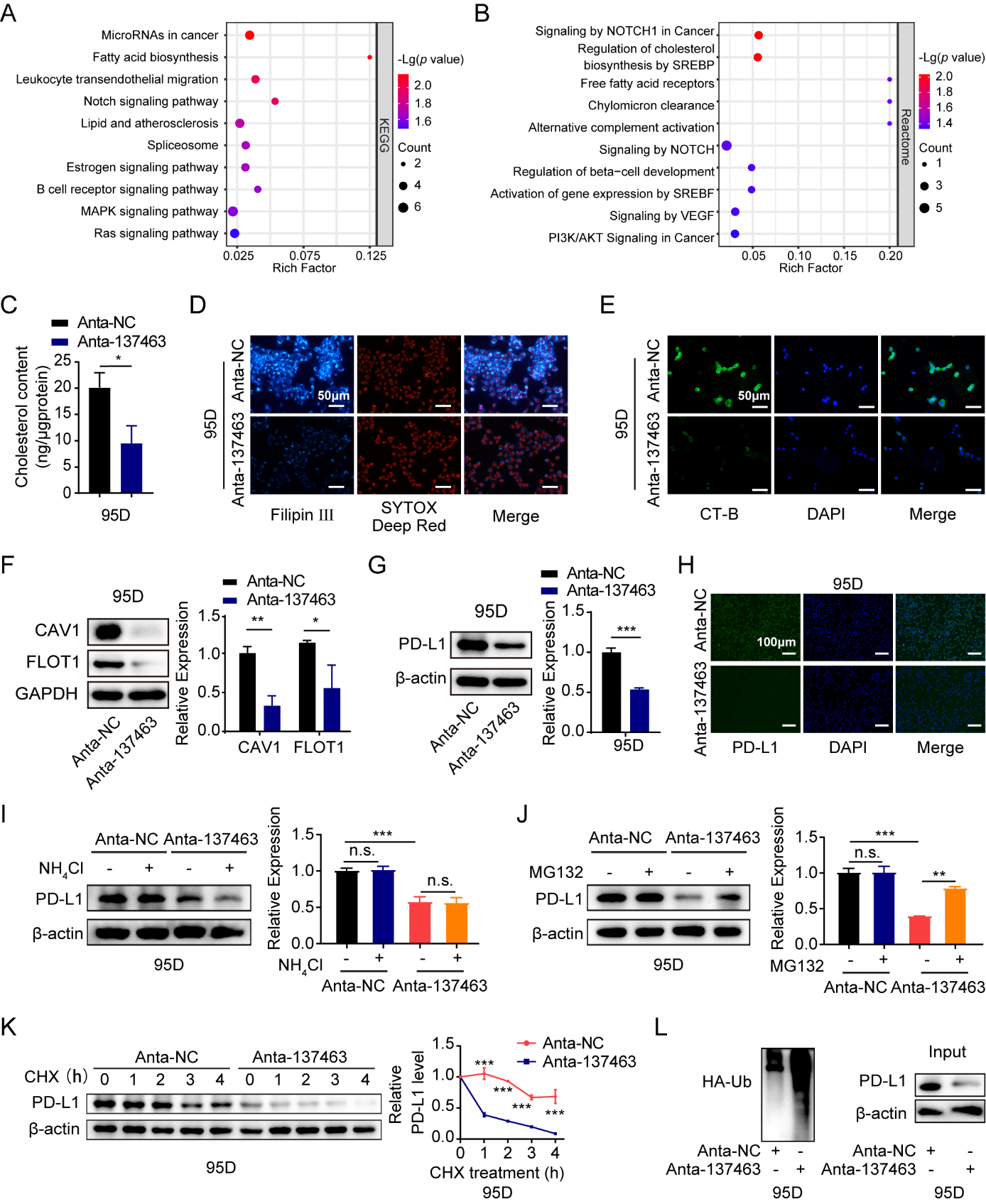


**Figure S3. piRNA-137463 regulates cholesterol metabolism, lipid raft** **content, and PD-L1 expression in 95D cells.** A) KEGG analysis of differentially expressed genes in piRNA-137463 downregulated cells. B) Reactome analysis of differentially expressed genes in piRNA-137463 downregulated cells. C) Effect of piRNA-137463 downregulation on cholesterol content in 95D cells. D) Filipin III staining of free cholesterol in 95D cells after antagopiR-137463 treatment. Nuclei were stained with SYTOX Deep Red. E) Effect of antagopiR-137463 on lipid raft content in 95D cells. Lipid raft content was indicated by fluorescence intensity of CT-B and nuclei were stained with DAPI. F) Immunoblotting of lipid raft markers in Anta-NC and Anta-137463. G, H) The effect of antagopiR-137463 on PD-L1 expression was detected by immunoblotting (G) and immunofluorescence (H). I, J) PD-L1 expression in Anta-NC and Anta-137463 was detected by immunoblotting after treatment with or without 10 mM NH_4_Cl (I) or 10 μM MG132 for 8 hours (J). K) The effect of piRNA-137463 inhibition on PD-L1 protein stability was investigated by CHX chase assays. L) AntagopiR-137463 enhanced the ubiquitin-mediated degradation of PD-L1. Cells were transfected with HA-Ub plasmid and treated with MG132 for 8 hours. The ubiquitination level of PD-L1 was detected by immunoprecipitation and immunoblotting. Data are presented as mean ± SD. Statistical analyses were performed using t-tests (C, F, and G) and two-way ANOVA (I-K). **P* < 0.05, ***P* < 0.01, ****P* < 0.001, *n.s.*, not significant.


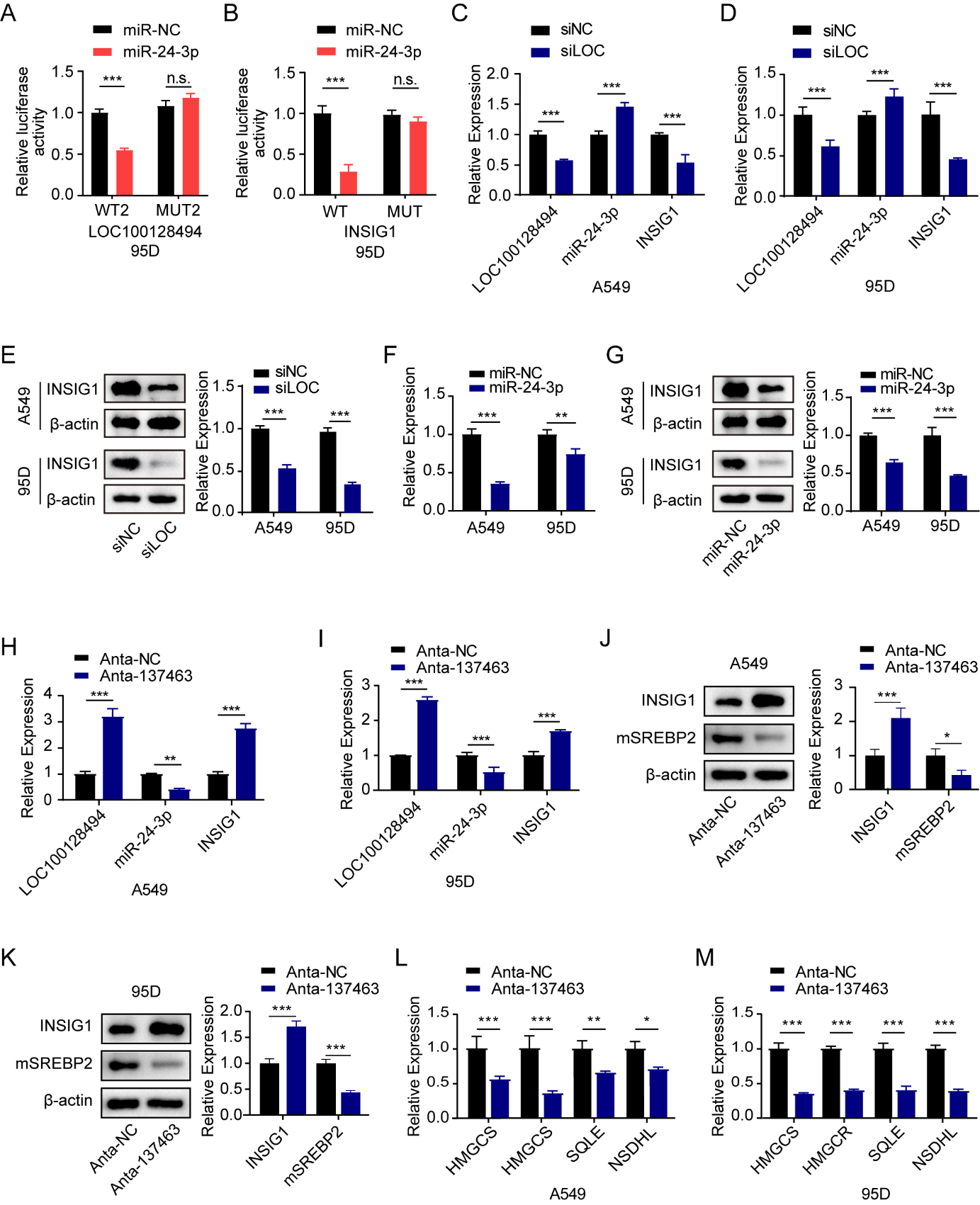


**Figure S4. Construction of LOC100128494/miR-24-3p/INSIG1 ceRNA network.** A) The interaction of miR-24-3p with LOC100128494 was verified by dual luciferase reporter assays in 95D cells. B) Interaction between miR-24-3p and INSIG1 verified by dual luciferase reporter assays in 95D cells. C, D) Effect of LOC100128494 on RNA expression of miR-24-3p and INSIG1 in A549 (C) and 95D (D) cells. E) Effect of LOC100128494 on protein expression of INSIG1 in A549 and 95D cells. F, G) Effect of miR-24-3p on the RNA expression (F) and protein expression (G) of INSIG1. H, I) The RNA expression of LOC100128494, miR-24-3p, and INSIG1 in Anta-NC and Anta-137463. J, K) INSIG1 and mature SREBP2 (mSREBP2) protein levels in Anta-NC and Anta-137463. L, M) The expression of genes downstream of m-SREBP2 involved in de novo cholesterol synthesis in Anta-NC and Anta-137463. Data are presented as mean ± SD. Statistical analyses were performed using *t*-tests. **P* < 0.05, ***P* < 0.01, ****P* < 0.001, *n.s.*, not significant.


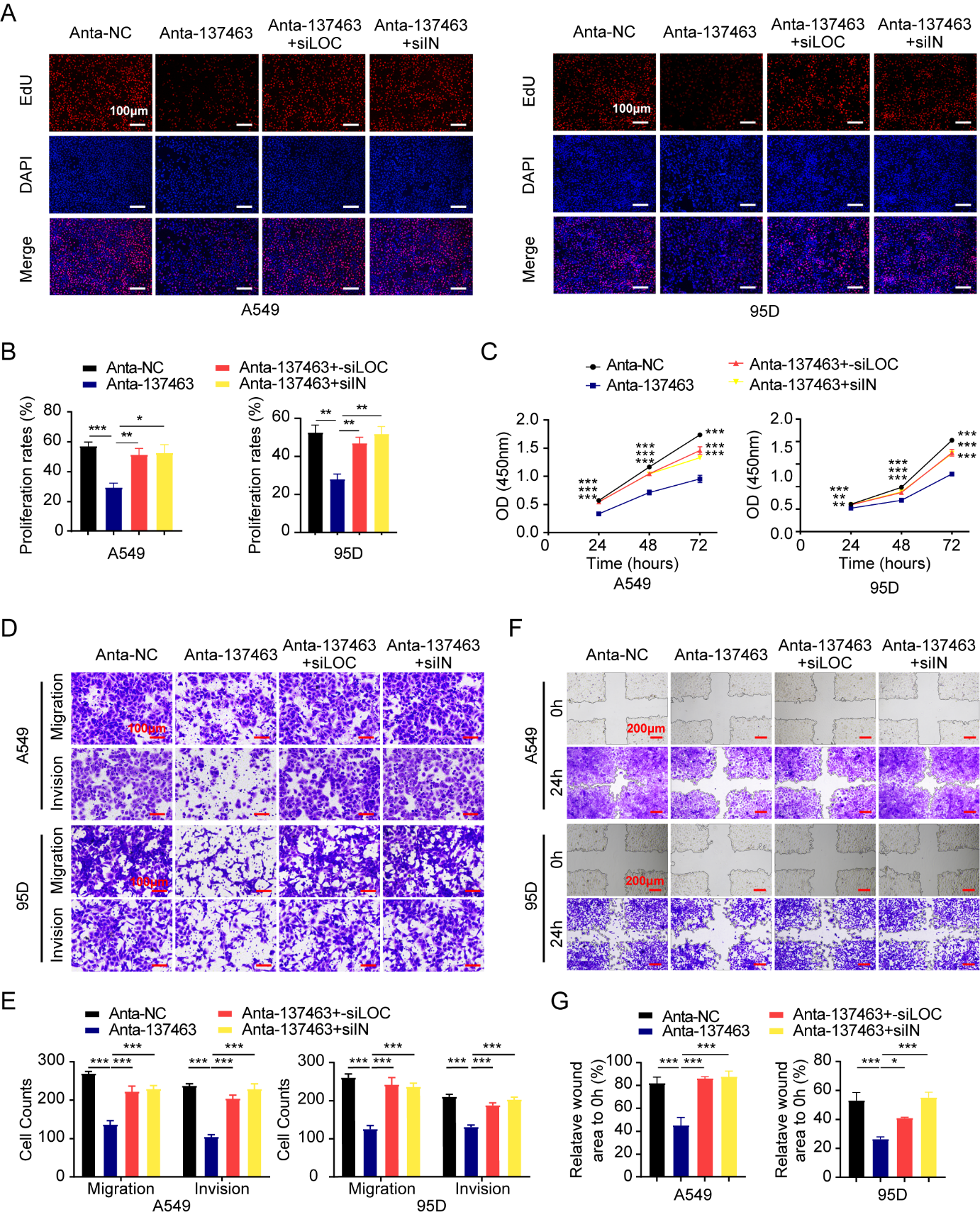


**Figure S5. piRNA-137463 influences cell proliferation, migration, and invasion in LUAD cells through LOC100128494 and INSIG1.** A) EdU incorporation assays were performed to assess the effect of LOC100128494 or INSIG1 knockdown on proliferative capacity following piRNA-137463 silencing. B) The bar plot shows the statistical analysis of EdU incorporation assays. C) CCK-8 assays illustrating cell viability across the designated groups. D, E) Crystal violet staining images (D) and statistical evaluation of migrating and invading cells (E) in the designated groups. F) Wound healing assays were conducted to assess the migration capability of LUAD cells. G) Bar plot showing the statistical analysis of wound healing assays. Data are presented as mean ± SD. Statistical analyses were performed using one-way ANOVA (B, E, and G) and two-way ANOVA (C). **P* < 0.05, ***P* < 0.01, ****P* < 0.001.


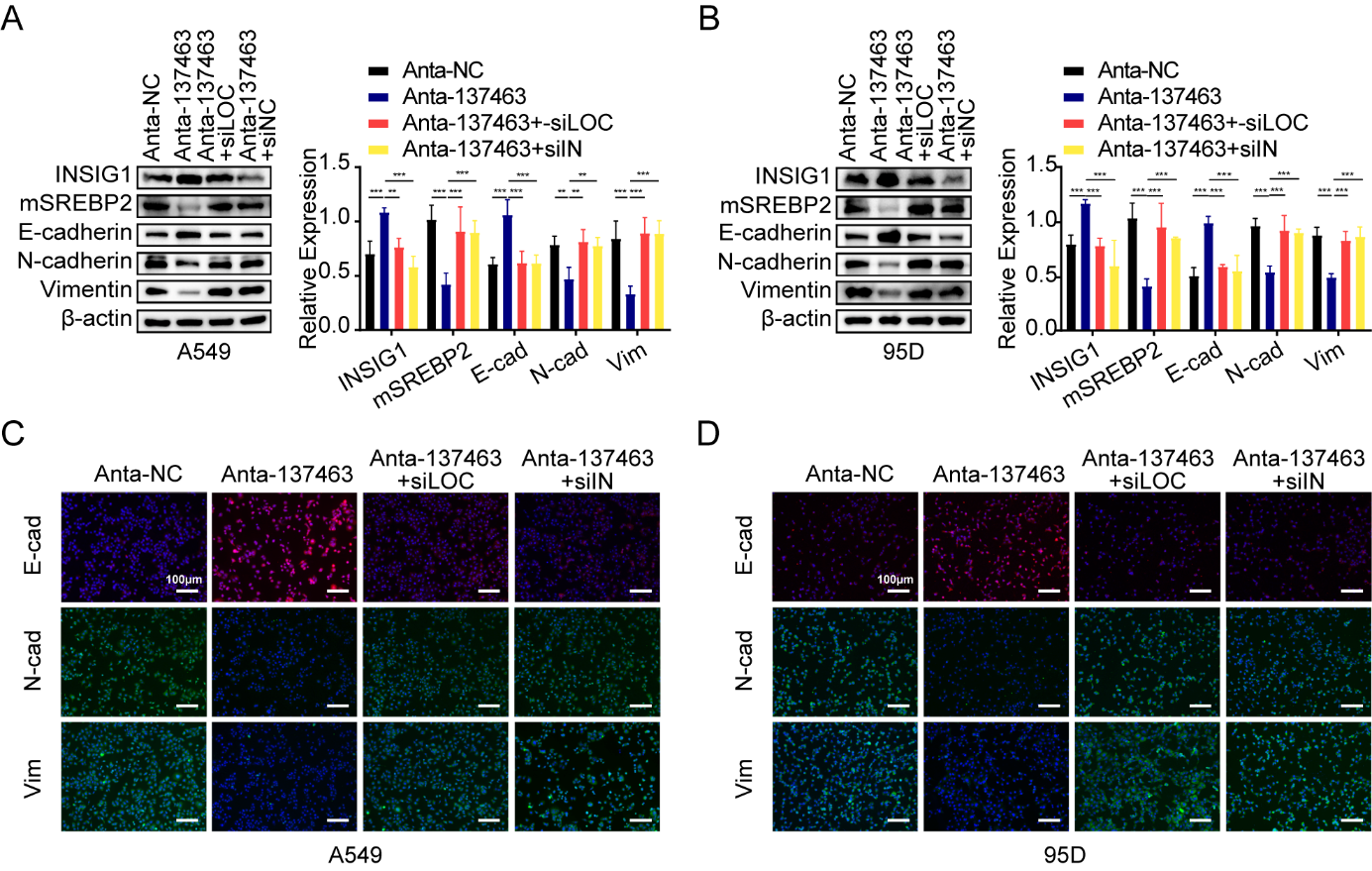


**Figure S6. piRNA-137463 mediates EMT in LUAD cells in a LOC100128494/INSIG1-dependent manner.** A, B) Immunoblotting was conducted to assess the impact of siLOC100128494 or siINSIG1 on EMT marker expression in LUAD cells following piRNA-137463 silencing. C, D) Immunofluorescence analysis was conducted to assess the impact of siLOC100128494 and siINSIG1 on E-cadherin, N-cadherin, and Vimentin protein expression in LUAD cells following piRNA-137463 silencing. Data are presented as mean ± SD. Statistical analyses were performed using one-way ANOVA (A and B). ***P* < 0.01, ****P* < 0.001, *n.s.*, not significant.


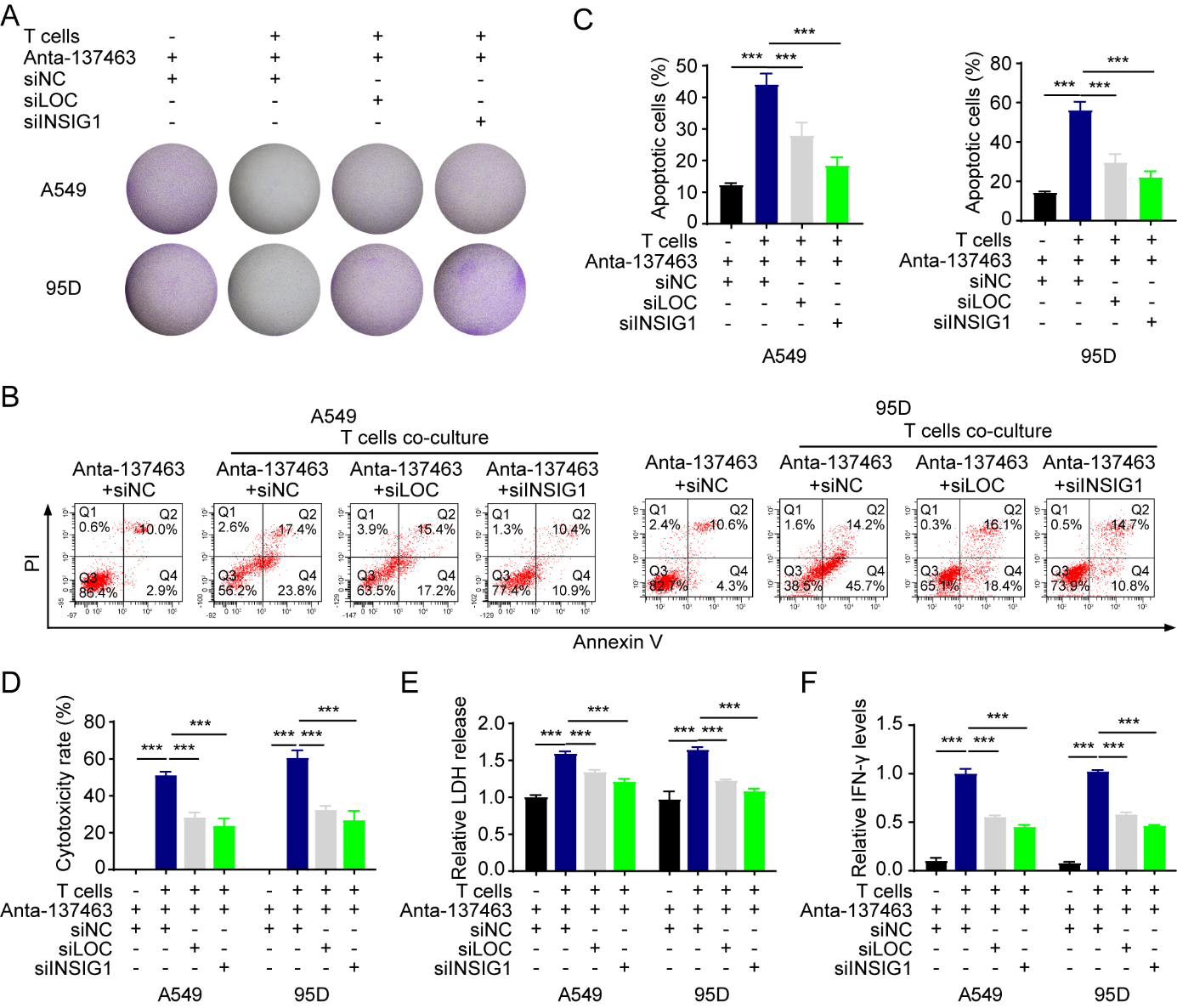


**Figure S7. piRNA-137463 promotes the immune escape of LUAD cells through LOC100128494 and INSIG1.** A) Crystal violet staining images of LUAD cells co-cultured with activated T cells in the indicated groups. B) T cell-mediated LUAD cell apoptosis was detected by flow cytometry in the indicated groups. C) Flow cytometry quantification of apoptotic cells. D) T cell-mediated cytotoxicity against LUAD cells in the specified groups. E) Levels of LDH released by LUAD cells co-cultured with T cells in the indicated groups. F) IFN-γ secretion by T cells co-cultured with LUAD cells in specified groups, measured via ELISA. Data are presented as mean ± SD. One-way ANOVA was used for statistical analysis (C-F). ****P* < 0.001.


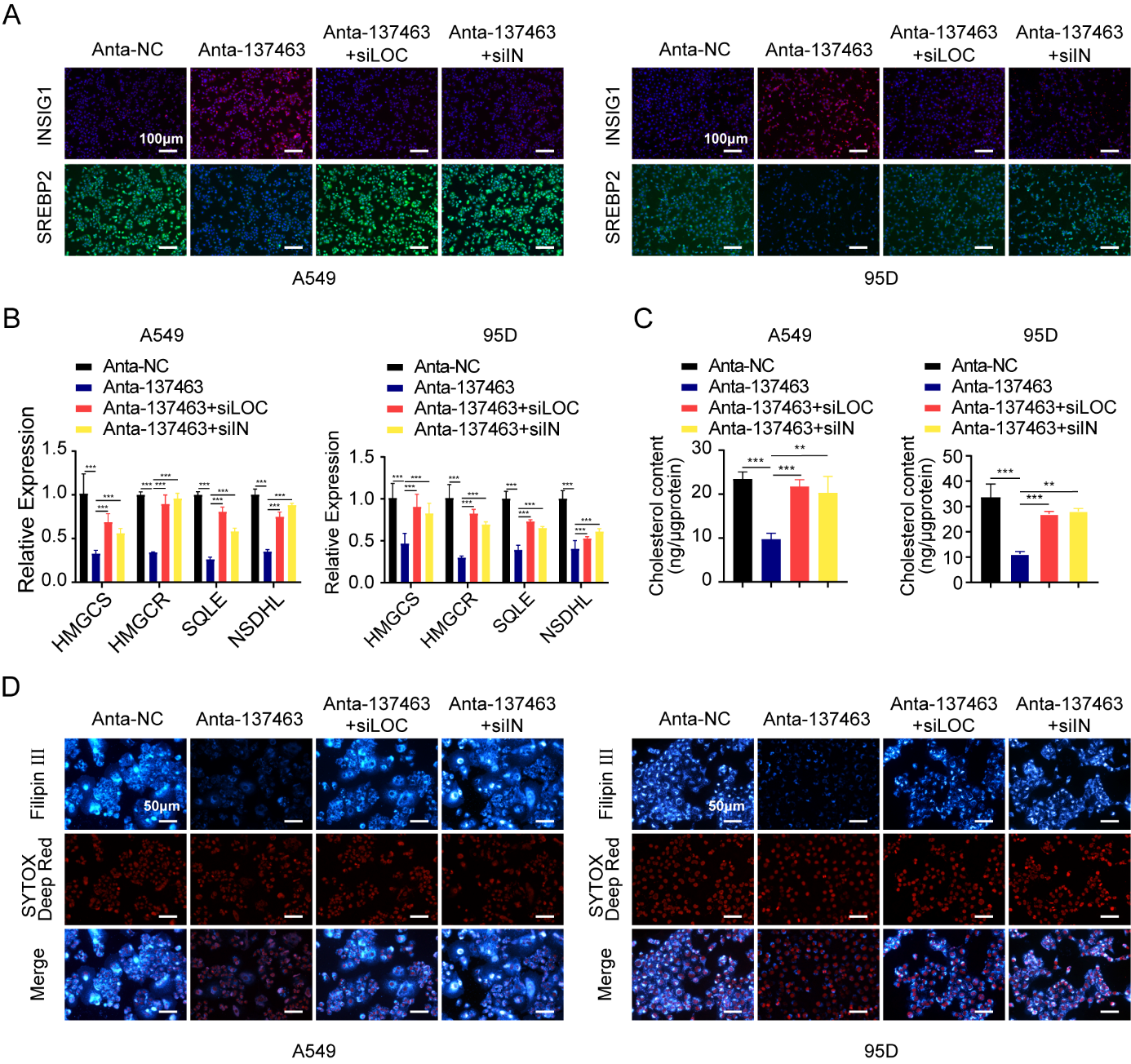


**Figure S8. piRNA-137463 regulates cholesterol levels via LOC100128494 and INSIG1.** A) Immunofluorescence images showing the effects of siLOC100128494 or siINSIG1 on INSIG1 and SREBP2 protein expression after piRNA-137463 silencing. B) qPCR analysis of the effect of siLOC100128494 and siINSIG1 on the transcript levels of SREBP2 target genes after piRNA-137463 silencing. C) Effects of LOC100128494 or INSIG1 knockdown on total cholesterol levels after piRNA-137463 silencing. D) Filipin III staining of free cholesterol in LUAD cells from the indicated groups. Nuclei were stained with SYTOX Deep Red. Data are presented as mean ± SD. Statistical analysis was performed using one-way ANOVA (B and C). ***P* < 0.01, ****P* < 0.001.


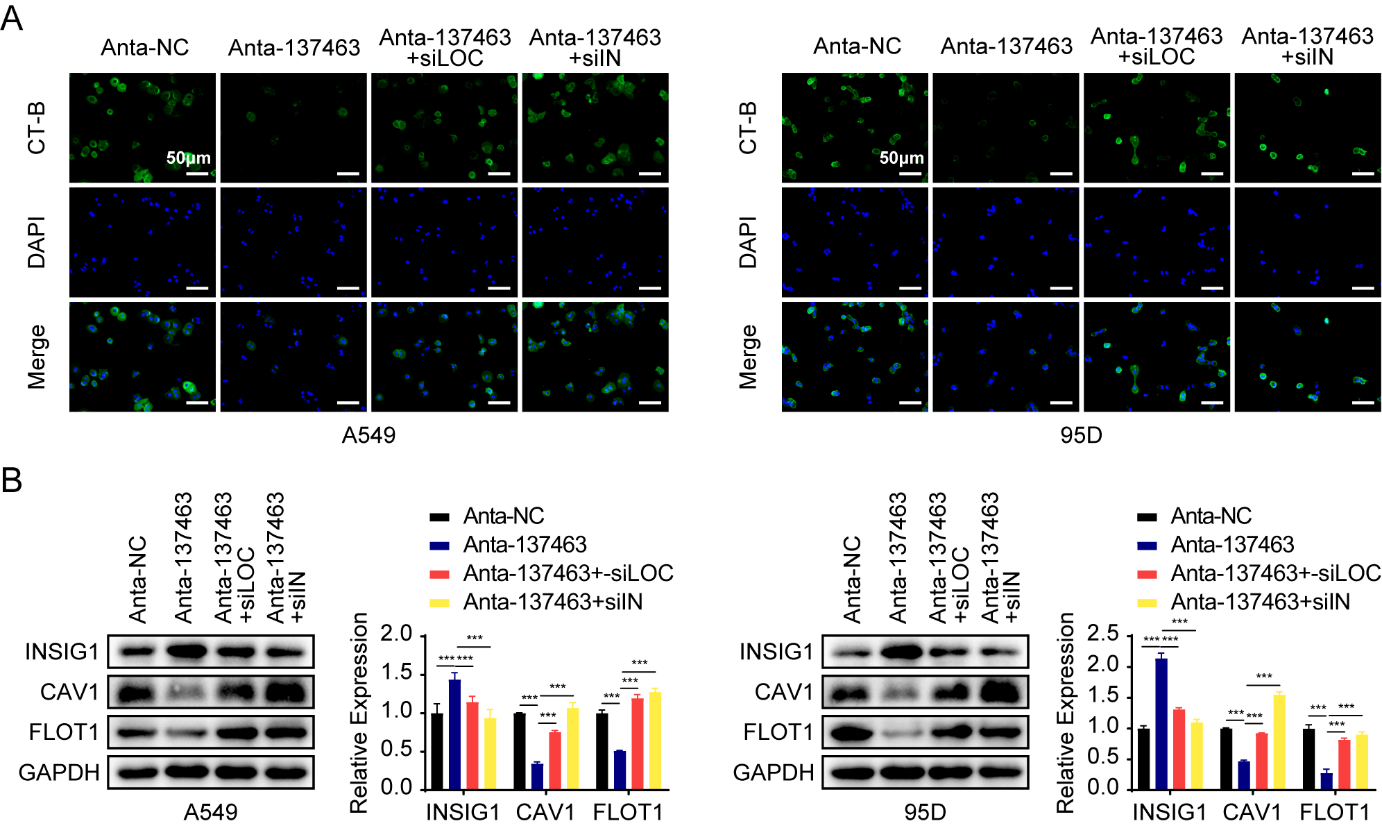


**Figure S9. piRNA-137463-mediated lipid raft content depends on LOC100128494 and INSIG1.** A) Effect of LOC100128494 or INSIG1 knockdown on lipid raft activity in antagopiR-137463-treated cells. Lipid raft activity was measured by the fluorescence intensity of CT-B with nuclei stained using DAPI. B) Effect of LOC100128494 or INSIG1 knockdown on lipid raft marker expression after piRNA-137463 silencing. Data are presented as mean ± SD. One-way ANOVA was used for statistical analysis. ****P* < 0.001.


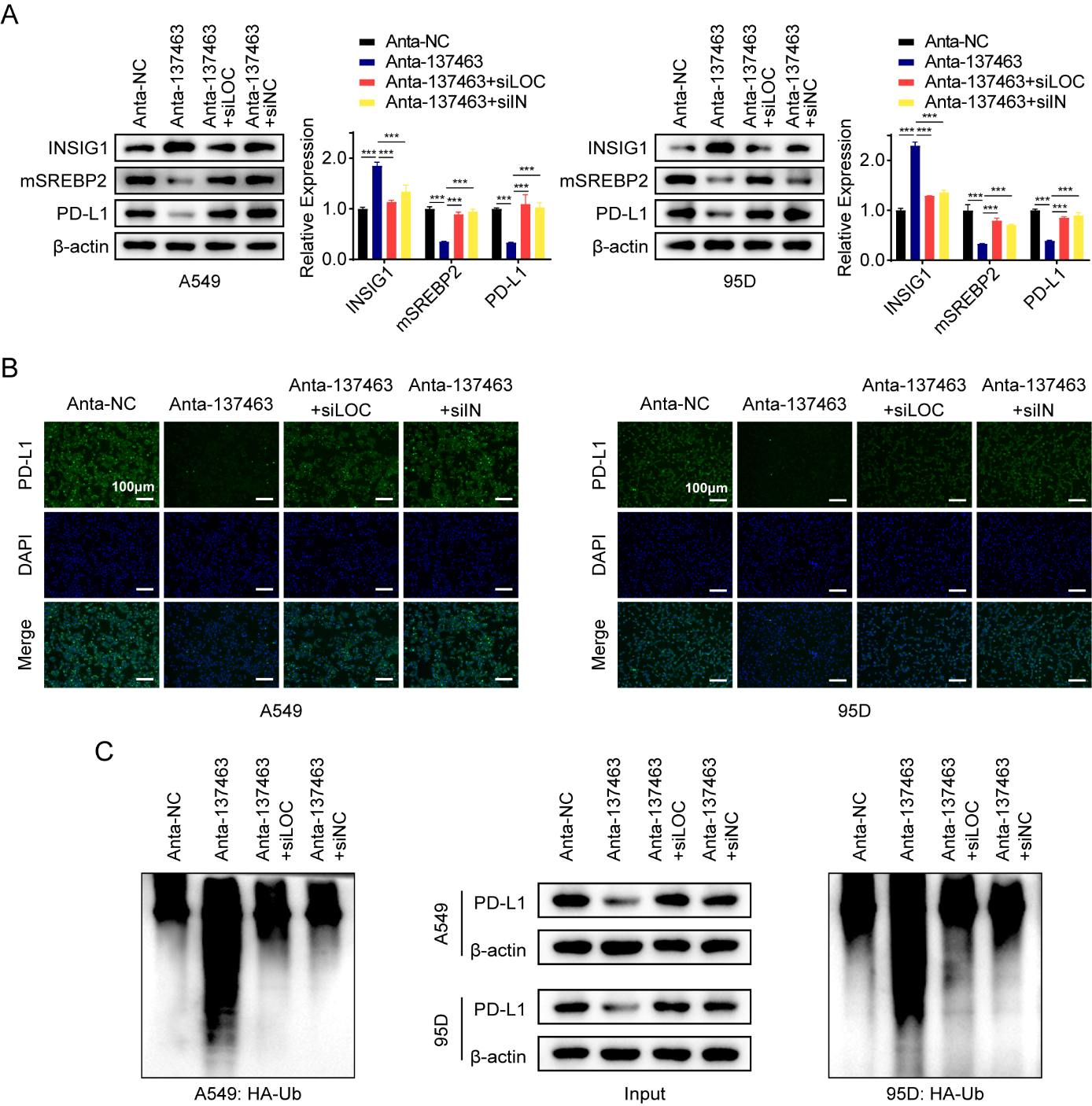


**Figure S10. piRNA-137463-mediated** **PD-L1 expression relies on LOC100128494 and INSIG1.** A, B) The effects of LOC100128494 or INSIG1 knockdown on PD-L1 expression after piRNA-137463 silencing were assessed using immunoblotting (A) and immunofluorescence (B). C) The effect of LOC100128494 or INSIG1 knockdown on the ubiquitinated degradation of PD-L1 after piRNA-137463 silencing was analyzed by ubiquitination immunoprecipitation. Data are presented as mean ± SD. One-way ANOVA was used for statistical analysis. ****P* < 0.001.


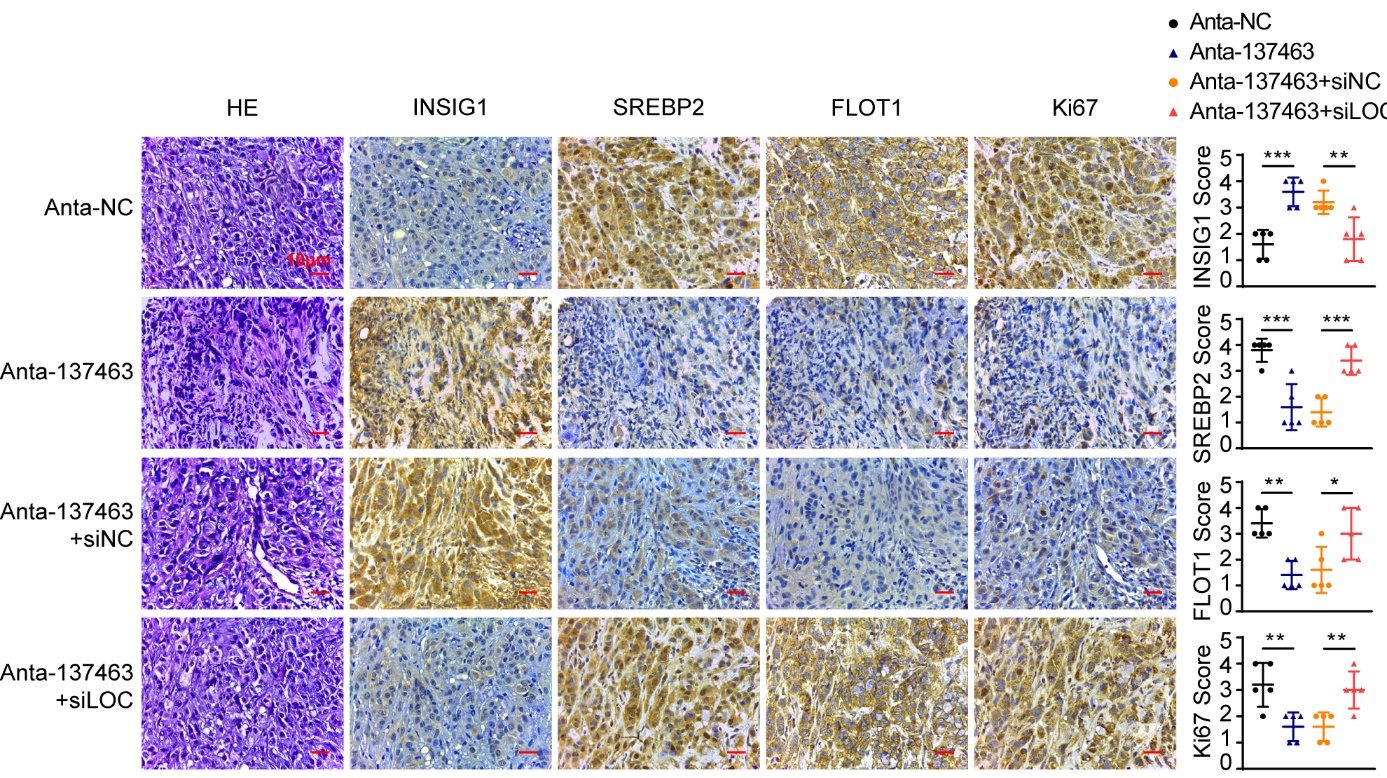


**Figure S11. Anta-137463 treatment inhibits tumor growth via LOC100128494 in nude mice.** Representative images of H&E staining and IHC for INSIG1, SREBP2, FLOT1, and Ki67 (left, along with IHC score statistics (right, from subcutaneous xenograft tumor models in the indicated groups. Data are presented as mean ± SD. Statistical analysis was performed using one-way ANOVA. **P* < 0.05, ** *P* < 0.01, *** *P* <0.001.


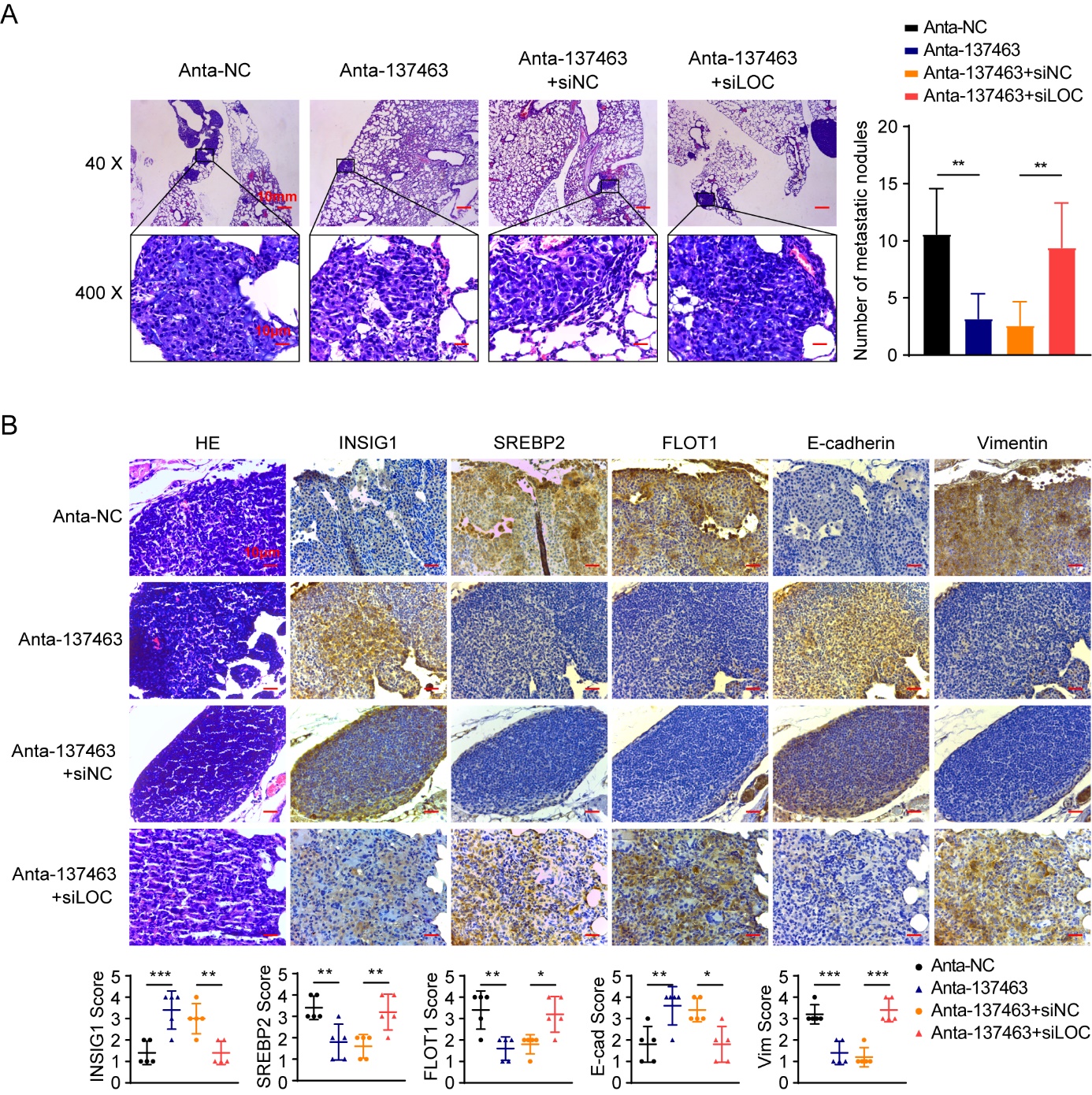


**Figure S12. Anta-137463 treatment inhibits tumor metastasis via LOC100128494 in nude mice.** A) Representative H&E images of lung tissues of nude mice in each group in the lung metastasis model (left) and statistics of the number of lung metastatic nodules in mice (right). B) Representative images of H&E staining and IHC (top) as well as IHC score statistics (bottom) for INSIG1, SREBP2, FLOT1, E-cadherin, and Vimentin in lung metastasis models from nude mice in the indicated groups. Data are presented as mean ± SD. Statistical analysis was performed using one-way ANOVA. **P* < 0.05, ** *P* < 0.01, *** *P* <0.001.
